# Biochemical Nanotubes Containing Heterocycles as Artificial Strands for Pseudo Duplex and Triplex DNA Formation

**DOI:** 10.1021/acs.jpcb.4c08079

**Published:** 2025-03-11

**Authors:** Jih Ru Hwu, Deepa Rohidas Landge, Wen-Chieh Huang, Jia-Cherng Horng, Yu-Chen Hu, Kuo Chu Hwang, Chun-Cheng Lin, Shwu-Chen Tsay

**Affiliations:** † Department of Chemistry, National Tsing Hua University, Hsinchu 30044, Taiwan; ‡ Frontier Research Center on Fundamental and Applied Sciences of Matters, National Tsing Hua University, Hsinchu 300044, Taiwan; § Department of Chemical Engineering, National Tsing Hua University, Hsinchu 300044, Taiwan

## Abstract

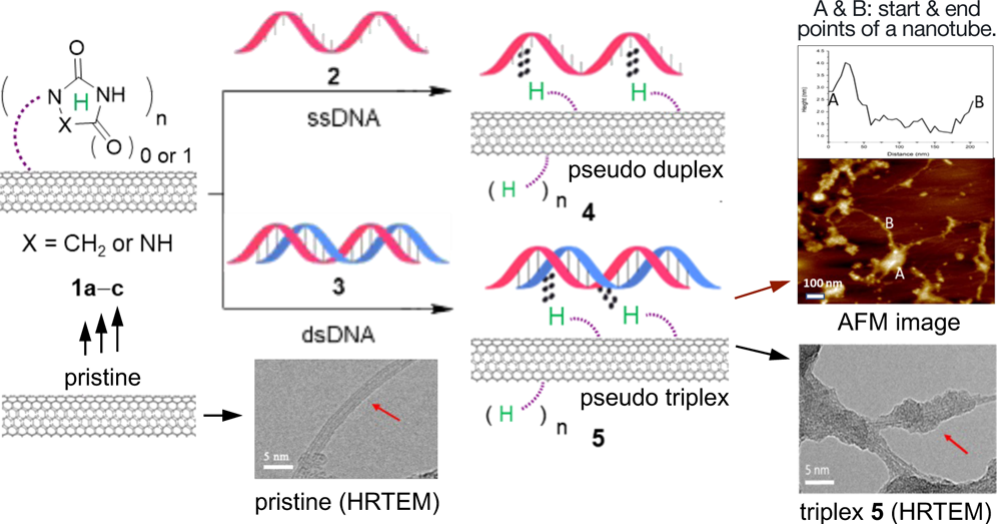

This report presents our discoveries that include the successful hybridization of grafted single-walled carbon nanotubes (SWCNTs) with dsDNA to form pseudo triplex-DNA. These tubes are attached with distinctive five-membered N-containing heterocycles (i.e., imidazolidinone and triazolidindione) on their surface. In this study, the heterocycles play a crucial role as DNA binders. Consequently, three functionalized SWCNTs (f-SWCNTs) are synthesized, which are incorporated with multiple–phenoxy–triazole–(ethylene glycol)–(heterocycle) ligands. These f-SWCNTs are entwined with dsDNA to form “pseudo triplex”. Notably, the dsDNA disengages from the f-SWCNTs at 85 °C and then is able to revert to triplex-DNA upon temperature reduction. Additionally, these f-SWCNTs act as a complementary strand for ssDNA to form pseudo duplex-DNA, in which the base pairings therein dissociate at 55 °C. Comprehensive analysis by use of CD spectrometer, SEM, TEM, and AFM microscopy provides substantive evidence for these phenomena. The demonstrated ability to manipulate DNA liberation from pseudo duplexes and triplexes indicates the potential versatility of f-SWCNTs as effective delivery vehicles for drugs and biomaterials in gene therapy and biotechnology.

## Introduction

Triple-stranded DNAs hold significant promise as biochemical materials for selectively inhibition of the expression of specific genes with a targeted DNA sequence.^1^ Their pharmacological applications in gene therapy are noteworthy, encompassing the ability to intervene disease-related genes, induce site-specific mutations, modulate gene expression, and more.^2^ Recent advancements in DNA-based nanomaterials, particularly triplex-DNA, have demonstrated utility in structural reconfiguration as functional nanomaterials.^3,4^ The formation of triple helical structures within different natural or artificial units can exert a profound impact on gene expression.^5^ While the delivery of a triplex-forming oligonucleotide (TFO) holds attractive applications,^6^ the energetically unfavorable nature of forming triple helical structures through Hoogsteen or reverse-Hoogsteen hydrogen bonding poses a notable challenge.^7^ Consequently, the applicability of triplex formation is primarily limited to a homopurine–homopyrimidine sequence in dsDNA.^8^

**Figure 1:**
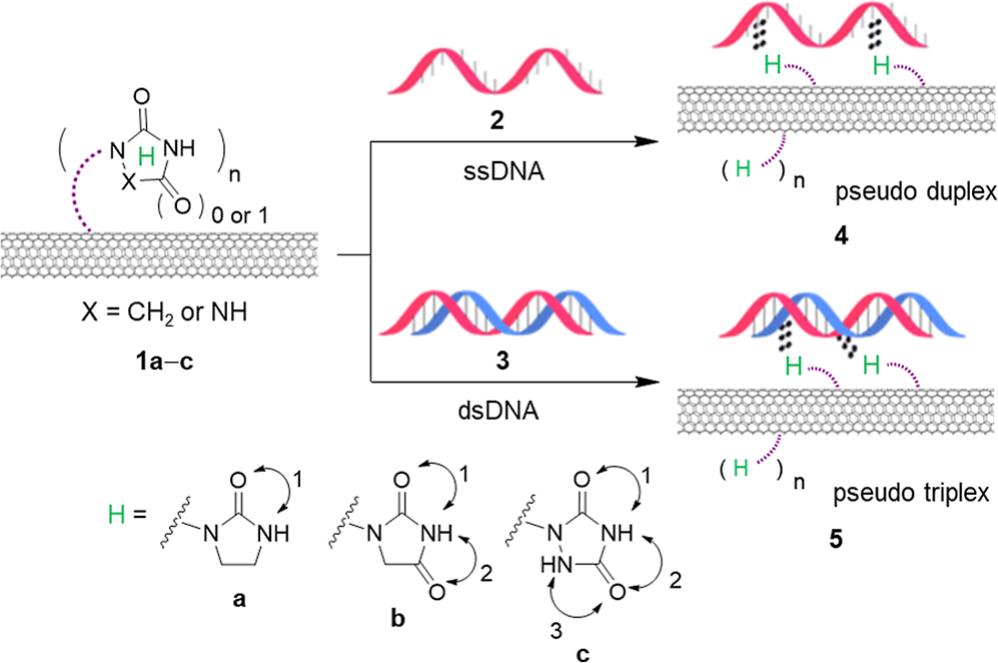
Formation of pseudo duplex-DNA **4** and triplex-DNA **5** by hybridization of f-SWCNTs **1a**–**c** with ssDNA **2** and dsDNA **3**, respectively. “H” represents the N-HDB with 1–3 binding sites (BS) as indicated by arc arrows: imidazolidine-2-one (**1a**, 1-BS), imidazolidine-2,4-dione (**1b**, 2-BS), and 1,2,4-triazolidine-3,5-dione (**1c**, 3-BS).

**Figure 2:**
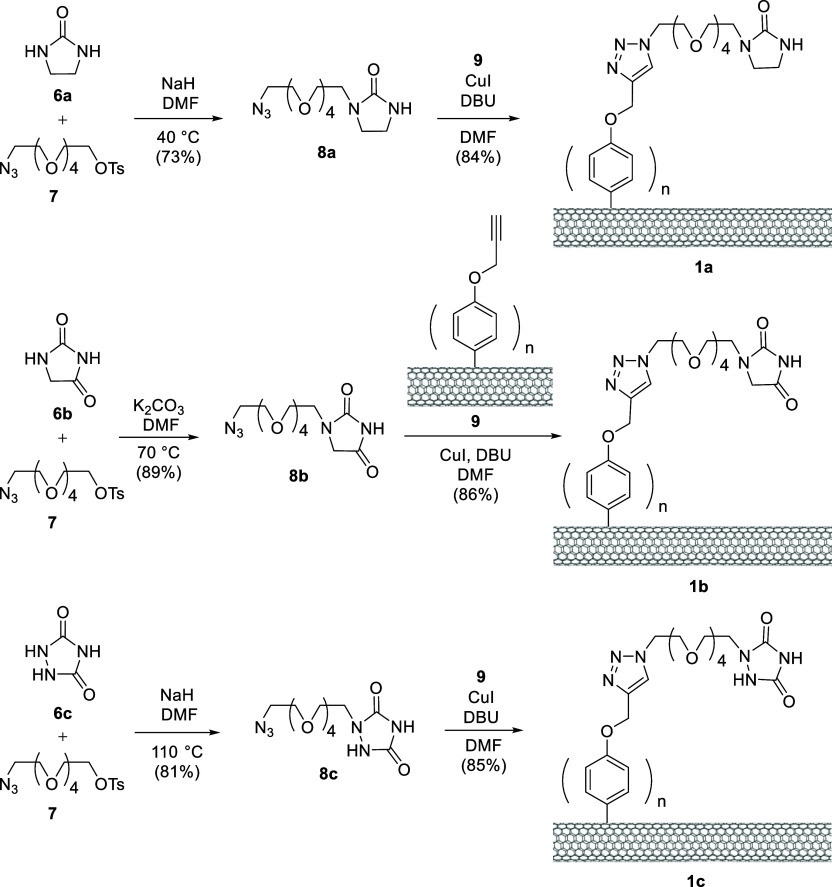
Synthesis of f-SWCNTs **1a**–**c** adorned with three different imidazolidinones or triazolidindiones as N-HDB at the terminal of ligands.

To overcome existing limitations, we propose an innovative design featuring the invention of biochemical rods as artificial TFOs, employing functionalized single-walled carbon nanotubes (f-SWCNTs). Multiple five-membered heterocycles, including imidazolidinones and a triazolidindione (i.e., **1a**–**c** in Figure 1), were designed to be grafted onto pristine SWCNTs via spacers. These nitrogen-containing heterocyclic DNA binders (termed N-HDB) were selected for their ability to form stable base pairs with the amino and carbonyl groups of nucleobases in DNA. The N-HDB requirements included both proton donor and proton acceptor functionalities at adjacent positions. These moieties, characterized by their small sizes, facilitated entry into the major or minor groove of dsDNA to form hydrogen bonds with nucleobases, such as Hoogsteen-type base pairings in triplex-DNA. The spacers, integral components of the design, comprised long ligands containing a hydrophobic phenoxy–triazole segment and a hydrophilic tetra(ethylene glycol) segment in **1a**–**c** (Figure 2). These ligands imparted flexibility to allow N-HDB to interact with dsDNA through hydrogen bonding. The amphipathic nature of the spacers contributed to the enhanced dispersibility of f-SWCNTs **1** in various solvents, including aqueous solutions.

Systematic investigations were conducted on the hybridization of f-SWCNTs **1a**–**c** with ssDNA **2** and dsDNA **3** to form the corresponding pseudo duplex-DNA **4** and triplex-DNA **5**, respectively (Figure 1). The results showed the capability of these biochemical rods (i.e., f-SWCNTs **1**) to mimic natural DNA strands. The invention of these f-SWCNTs, adorned with multiple imidazolidinones or triazolidindiones, introduced different features and applications to the realms of biotechnology and biomedicine.

Various DNA insertors belonging to five-membered N-heterocycles have emerged as promising candidates for application owing to their capacity to insert into DNA base pairs, disrupt original hydrogen bonds, and establish fresh hydrogen bonds with DNA nucleobases.^9^ In this study, a binding site (BS) is defined as a pair of proton donor and acceptor groups within the binder. A single five-membered N-heterocyclic compound may harbor up to three binding sites. Examples of such compounds with 1–3 BS include imidazolidine-2-one (1-BS), imidazolidine-4,5-dione (1-BS), pyrazolidine-3,5-dione (1-BS), imidazolidine-2,4-dione (hydantoin, 2-BS), imidazolidine-2,4,5-trione (parabanic acid, 2-BS), 1,2,4-oxadiazolidine-3,5-dione (2-BS), 1,2,4-triazolidine-3,5-dione (urazole, 3-BS), 1,2,4-triazolidine-3,5-dithione (3-BS), and others. Among these candidates, imidazolidine-2-one, hydantoin, and urazole were selected for modification and subsequent attachment to pristine SWCNTs (as illustrated in Figure 1), chosen for their commercial availability, cost-effectiveness, and representation of N-HDB with 1–3 BS. A binder featuring more binding sites increases the likelihood, resulting from different orientations, of binding to the base pairs of DNA. Consequently, f-SWCNTs **1a**–**c** were identified as our targeted materials for the formation of pseudo duplex- and triplex-DNAs.

In this report, we present hybridizations of N-HDB-grafted SWCNTs **1** with ssDNA and dsDNA. It leads to the corresponding pseudo double- and triple-stranded DNAs **4** and **5**, respectively (see Figure 1). Our findings underscore that these f-SWCNTs **1** function as ideal artificial DNA strands for gene complexation. This approach introduces promising materials for potential applications in gene therapy and gene research.

## Experimental Methods

### Circular Dichroism (CD) Measurement

The CD spectra of DNA hybrids **4a**–**c** and **5a**–**c** in a sodium phosphate buffer were recorded on a CD spectrometer (model 410, AVIV Biomedical Inc.). Before measurement, the samples were placed in rectangular quartz glass cuvettes (Hellma GmbH & Co. KG) with a 1.00 mm optical path length at 25 °C. For prevention of ozone formation and minimization of damage to the optical system, the instrument was purged with N_2_ gas to remove O_2_ from the lamp housing and sample compartment. DNA hybrids **4a**–**c** and **5a**–**c** (0.40 mL each) were pipetted into the cuvette, and measurements were taken over a spectral range of 400–200 nm, with a scan rate of 10.0 nm/min and a spectral width of 2.00 nm. Additionally, ssDNA and dsDNA were measured for comparison. All data were analyzed by use of Aviv Biomedical Inc. and Origin software.

### Scanning Electron Microscopy Measurement and Sample Preparations

Scanning electron microscopy (SEM) images were obtained by use of an SEM spectrometer (JEOL JSM-7000F) operated at 5–15 kV and a working distance of 10.0 mm. Functionalized SWCNTs **1a**–**c** were dispersed in water (5.0 μg/mL) through ultrasonication for 10 min. A portion of this dispersed solution (4.0 μg/mL) was drop-casted onto a silicon wafer and dried at 40 °C.^10^ The sample was washed twice with 70% ethanol for 2.0 min each, followed by a wash with DI water for 1.0 min. The sample was left to dry at room temperature. A similar procedure was used for the DNA hybrids **4a**–**c** and **5a**–**c** (5.00 μL), which were deposited on a Si/SiO_2_ silicon wafer (2.00 mm × 2.00 mm) and dried at room temperature. The wafers were coated with a thin layer (0.50 nm) of platinum by use of a sputtering technique (JEOL, JFC-1600 Auto fine coater) at 10.0 mA for 13 s.^11^

### Transmission Electron Microscopy Measurement and Sample Preparations

The diameters and morphology of the functionalized SWCNTs **1a**–**c** and their corresponding DNA hybrids **5a**–**c** were examined by use of a JEOL JEM-2100 microscope at accelerating voltage of 200 kV. Sample preparation involved the use of a diluted dispersion of each sample drop-casted 1.0 μL onto a Lacey Formvar/Carbon copper grid (200 mesh size).^12^ All samples were air-dried overnight before imaging. The resultant images were analyzed by use of image analysis software.

### Atomic Force Microscopy Measurement and Sample Preparations

Tapping-mode atomic force microscopy (AFM) measurements were performed in air by use of a Bruker Dimension Icon system (Bruker Instruments). The system was operated under ambient conditions with phosphorus doped silicon tips (Olympus OMCL, resistivity 0.01–0.02 Ω/cm, length 160 μm, width 30.0 μm, normal spring constant 26 N/m, and resonance frequency 240–300 kHz). The AFM provided a resolution of *xy* < 0.150 nm and *z* ≈ 0.035 nm. For sample preparations, a droplet of poly-l-lysine solution (1.0%), freshly diluted with deionized (DI) water, was deposited on freshly cleaved mica (muscovite) for 15 min, then extensively rinsed with water and dried under nitrogen.^13^ Samples **4a**–**c** and **5a**–**c** were filtered through an Amicon Ultra-0.5 Centrifugal (Merck, Millipore Ltd.) unit with a 30,000 molecular weight filter three times at 14,000 rpm for 30 min each to remove free DNA. To recover the hybrid, buffer (PBS) or DI water (0.10 mL) was added and then reverse-spin filtered at 1000 rpm for 5.0 min. The recovered DNA@f-SWCNT was adjusted to a final volume of PBS buffer (0.40 mL) or DI water. For AFM imaging, each sample (i.e., **4a**–**c**, 10.0 μL for each) was deposited on the mica and allowed to adsorb on the surface for 5.0 min. The mica surface was then gently rinsed with DI water three times and dried under a stream of nitrogen. Functionalized SWCNTs **1a**–**c** and **5a**–**c** samples were dispersed in water (5.0 μg/mL), deposited on silicon wafer (2.00 mm × 2.00 mm), and dried at room temperature. The images were analyzed by use of NanoScope Analysis and ImageJ software.

## Results and Discussion

### Synthesis of N-HDB-Attached SWCNTs

Three synthesis routes were employed to produce f-SWCNTs **1a**–**c** as depicted in Figure 2. These routes shared common steps involving the N-alkylation of nitrogen-containing heterocycles, namely imidazolidine-2-one (**6a**), imidazolidine-2,4-dione (**6b**), and 1,2,4-triazolidine-3,5-dione (**6c**), with azido tosylate **7**. In the presence of NaH and K_2_CO_3_ in DMF, the corresponding **8a**, **8b**, and **8c** were obtained in 73–89% yields. Subsequently, the click reaction was applied for the preparation of N-HDB-attached SWCNTs **1a**–**c** by treatment of (phenoxy)alkynated SWCNT **9**(14) (with diameters of 1–2 nm and lengths of 5–30 μm) with azides **8a**–**c**. These organic compounds (1.0 equiv) were added to a dispersed DMF solution containing SWCNT **9** (31.7 mg), CuI (5.5 equiv), and 1,8-diazabicycloundec-7-ene (DBU, 14 equiv) at 110 °C for ∼4 days. The resultant paste was diluted with DMF at 25 °C, filtered through a poly(tetrafluoroethene) membrane, and the collected solids were washed with DMF and THF sequentially. The solids were then dispersed in *N*-methyl-2-pyrrolidone (NMP) by sonication for 20 min. After standing for 2–3 min, the less dispersible alkynated SWCNT **9** settled down. The supernatant was carefully filtered and the solids on the membrane were collected, washed with dichloromethane to remove NMP. The remaining solids were dried at 40 °C to give the desired triazole-containing (poly *N*-HDB)-SWCNTs **1a**–**c** (40.5–41.6 mg, charcoal black) in 84–86% yields. Detailed synthetic procedures, structural characterization, and physical properties of f-SWCNTs **1a**–**c** by use of NMR, IR, Raman, TGA, CD, and HR-Mass spectrometers are provided in the Supporting Information.

### Radial Breathing Mode and Degree of Covalent Functionalization of f-SWCNTs **1**

**Figure 3:**
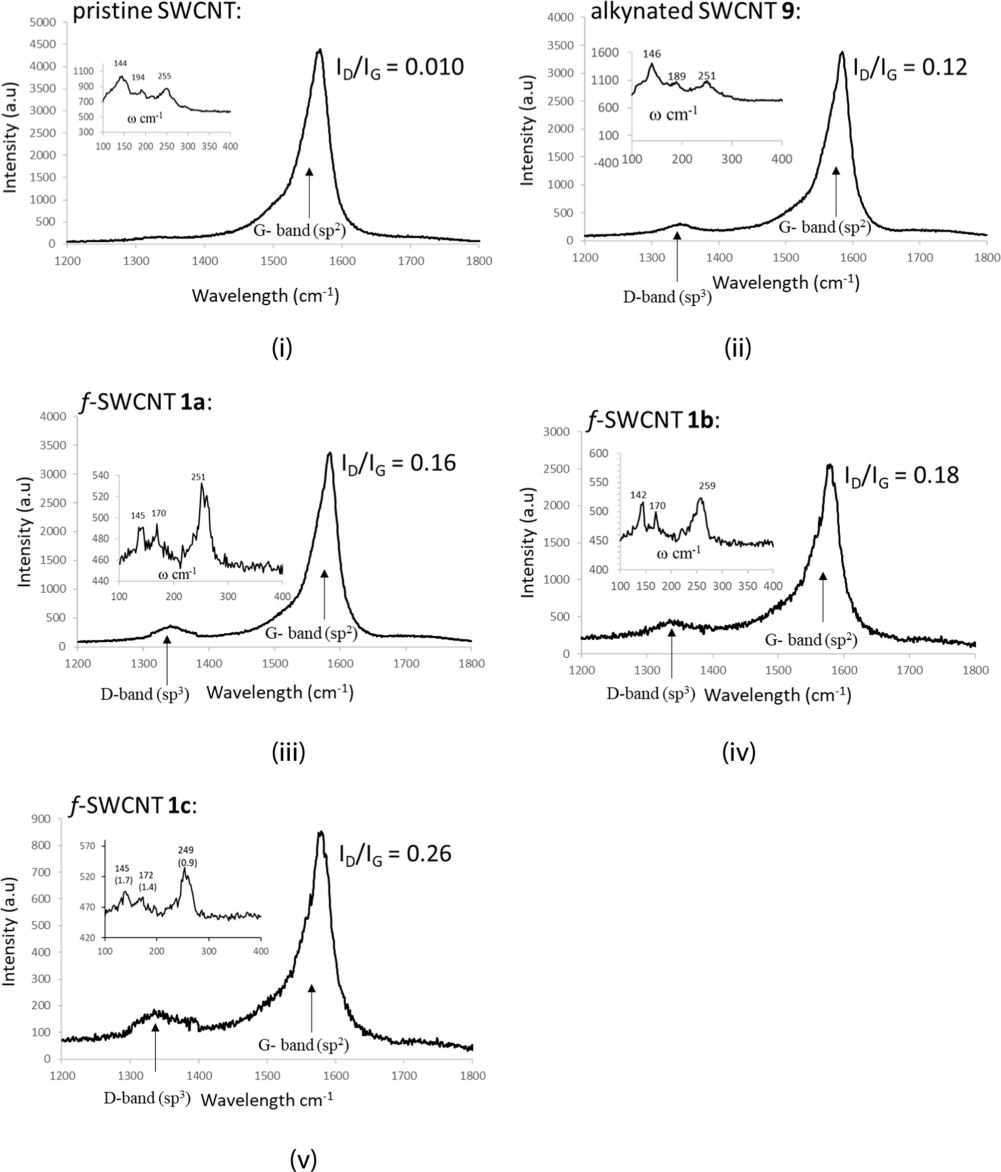
Raman spectra of various SWCNTs, their G- and D-band absorptions, *I*_D_/*I*_G_ values, and their radial breathing mode.

The Raman spectra revealed the emergence of D-band absorptions in f-SWCNTs **1a**–**c**, which were not observed in pristine SWCNT. The D-bands serve as a fingerprint to confirm the functionalization of the pristine SWCNTs and the alternation of their carbon network from sp^2^ → sp^3^.^15,16^ Additionally, the intensity ratios of the D band (*I*_D_) to the G band (*I*_G_) increased from 0.010 in pristine SWCNT to 0.16–0.26 in f-SWCNTs **1a**–**c**. The diameters of these samples were calculated by use of the radial breathing mode (RBM) frequencies (Figure 3 and Table 1).^17,18^ The calculated diameters (0.93–1.77 nm) fell into three ranges for the diameters of the central carbon nanotube networks, excluding the peripheral ligands^19^ in **9** and **1a**–**c**. These data indicate that the density of defects in **1a**–**c** increased after the functionalization of the pristine SWCNT.

**Table 1:** Calculated SWCNT Diameters on the Basis of the Measured Radial Breathing Mode Frequencies and Their *I*_D_/*I*_G_ Values

SWCNT	RBM frequency (cm^–1^)	calculated diameter (nm)	*I*_D_/*I*_G_
pristine	144, 194, 255	1.77, 1.27, 0.95	0.010
alkynated **9**	146, 189, 251	1.72, 1.30, 0.97	0.12
**1a**	142, 170, 251	1.72, 1.46, 0.97	0.16
**1b**	145, 172, 249	1.73, 1.44, 0.98	0.18
**1c**	142, 170, 259	1.72, 1.46, 0.93	0.26

**Figure 4:**
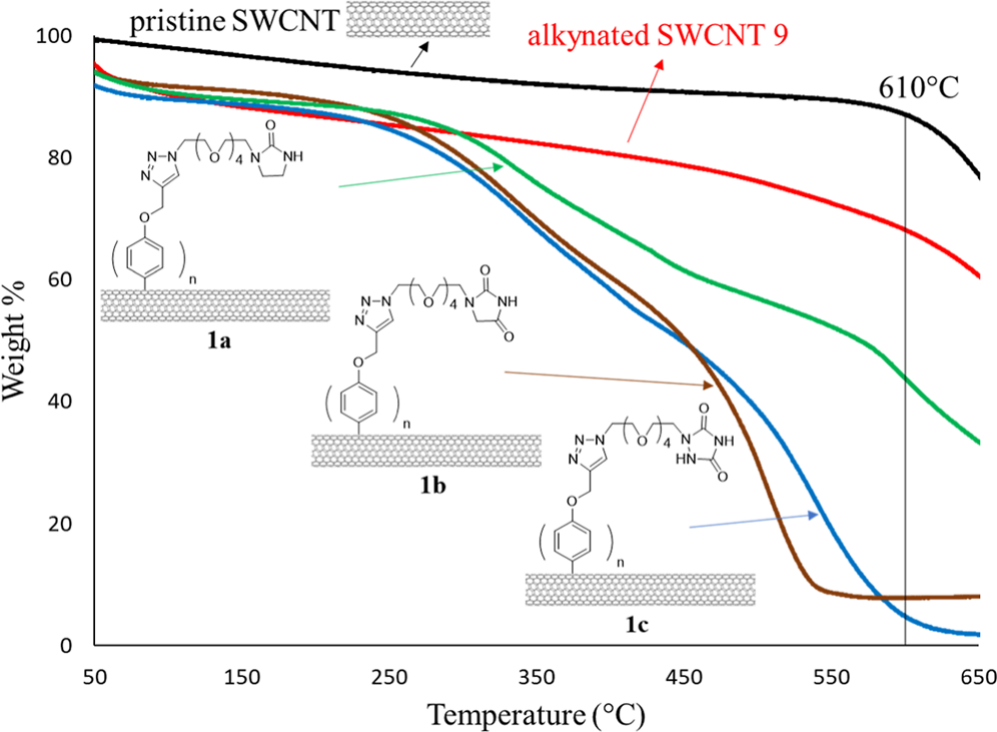
Thermogravimetric analysis of f-SWCNTs **1a**–**c** and **9**, along with the pristine nanotube under a temperature increment of 10 °C/min.

The degree of functionalization for the nanotube samples was investigated by use of thermogravimetric analysis (Figure 4). Different shapes of curves associated with respective mass changes in their thermograms clearly indicate that these N-HDB-attached ligands were successfully grafted to the pristine SWCNT. The functionalization degree of alkynated SWCNT **9** was estimated by use of Campidelli’s method, with an average of one (phenoxy)alkynyl ligand per 41 carbon atoms (see Figure S1 in the Supporting Information).^14,20^ The click reactions shown in Figure 4 were 84%, 86%, and 85% yields for the formation of f-SWCNTs **1a**–**c**, respectively. Thus, it allowed us to determine the number of N-HDB-attached ligands in f-SWCNTs **1a**–**c** with an average of one ligand per 49, 47, and 48 carbon atoms, respectively.

### Hybridization of DNA with f-SWCNTs 1a–c

The obtained (poly N-HDB)-SWCNTs **1a**–**c** were individually hybridized with ssDNA **2** and dsDNA **3** to form pseudo duplex-DNA **4** and triplex-DNA **5**, respectively. For instance, the practicability of SWCNT **1c** was demonstrated by dispersion (1.02 mg) in sodium phosphate-buffered saline (50 mM NaPBS, 0.501 mL), which contained a 25-mer of ssDNA **2** (5′-TCGAGTACGTCGCCGTCCAGCTCGA-3′, 0.502 mM, 0.480 mg). After sonication (3 W) at 0 °C for 90 min, the solution was centrifuged at 13000 rpm for 90 min to eliminate insoluble material. At this stage, all of ssDNA **2** was utilized, and the resultant hybrid duplex **4c** (i.e., ssDNA **2**@SWCNT **1c**) was obtained and found to be soluble in an aqueous solution. This duplex **4c** was analyzed by use of circular dichroism (CD) spectroscopy. The same procedure was also applied to f-SWCNTs **1a** and **1b**. Furthermore, the hybridization practicability between SWCNTs **1a**–**c** with and a 25-mer of dsDNA **3** (5′-TCGAGTACGTCGCCGTCCAGCTCGA-3′ and its complementary strand, 0.502 mM, 0.480 mg) to form pseudo triplex-DNA **5a**–**c** was tested. Similar positive results as described above were observed.

### CD Studies of Pseudo Duplex-DNA 4 and Pseudo Triplex-DNA 5

#### Binding Efficiency between DNA and f-SWCNTs **1a–c**

**Figure 5:**
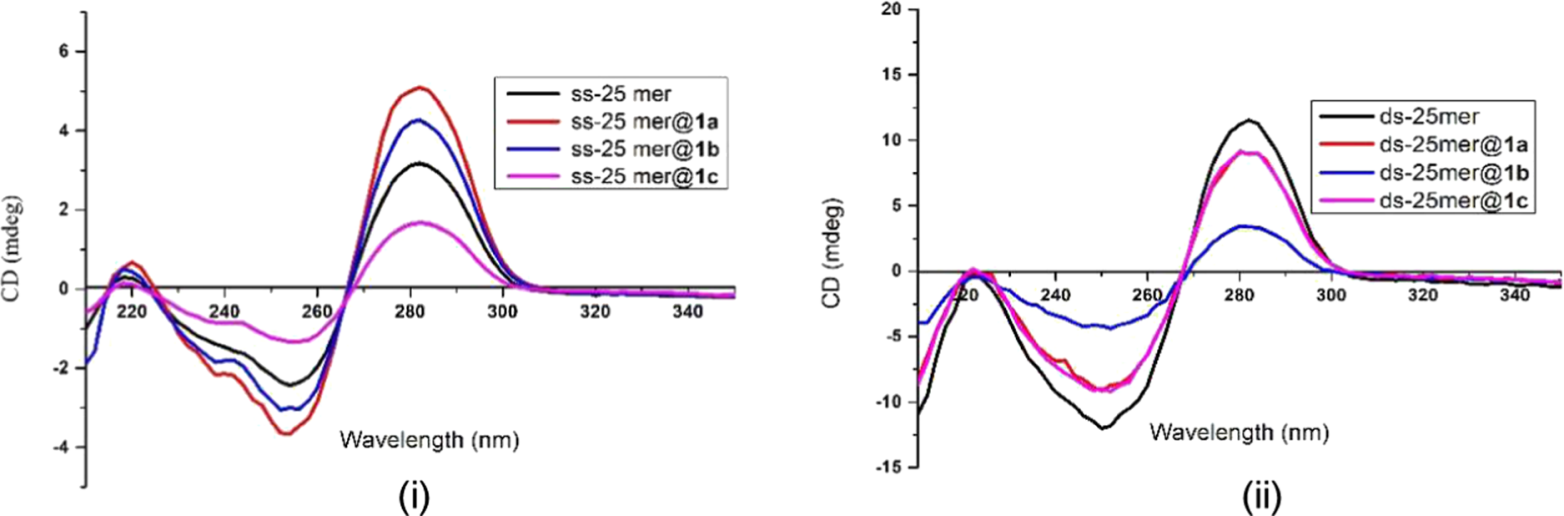
CD spectra of f-SWCNTs **1a**–**c** entwined in a buffer solution of pH 7.4 at 25 °C by (i) ssDNA **2** (i.e., ss-25 mer) to give pseudo duplexes **4** (i.e., ss-25 mer@**1**), and (ii) dsDNA **3** (i.e., ds-25 mer) to give pseudo triplexes **5** (i.e., ds-25 mer@**1**). Each set contained a free ssDNA **2** or dsDNA **3** as the references.

CD spectroscopy serves as a robust tool for investigation into the complexation and temperature effect on the secondary structures and conformation of nucleic acids. The binding efficiency analysis between DNAs and f-SWCNTs relies on scrutinizing changes in absorbance intensity (molar ellipticity [θ] = mdeg) and wavelength shifts (Δλ) in CD curves.^21,22^ These alternations provide valuable insights into the structural modifications occurring during the interaction. In Figure 5i, notable changes were observed in the CD intensities of both positive and negative bands for ssDNA **2**@f-SWCNTs **1** (i.e., pseudo duplex **4**) in comparison to the parent ss-25-mer **2**. Upon hybridization of f-SWCNTs **1a** and **1b** with ssDNA **2**, there was a discernible increase in CD intensities for both positive and negative peaks. Remarkably, **1a** induced a more pronounced CD intensity increment than **1b**. Conversely, the hybridization of **1c** with ssDNA resulted in reduction of CD intensities for **4c** due to a diminished the base–base interaction.^23^ Particularly noteworthy was a 2.0 nm hypsochromic shift of the negative band and no discernible change in the positive band for **4c** (pink line).

The impact of carbon nanotubes (i.e., f-SWCNTs **1a**–**c**) on the CD spectra, however, was found to be negligible, as illustrated in Figure S2 in the Supporting Information. Therefore, the discernible spectral variations in ssDNA **2** were primarily ascribed to its interaction with the f-SWCNTs **1a**–**c**.^24^ The CD results clearly demonstrate the binding capability of **1a**–**c** to ssDNA **2**.^22^

As depicted in the CD spectra of Figure 5ii, all three f-SWCNTs **1a**–**c** successfully hybridized with dsDNA **3** to form pseudo triplexes **5**. The dsDNA **3** used was a 25-mer with a sequence of 5′-TCGAGTACGTCGCCGTCCAGCTCGA-3′ bound to its complement. In the B-form dsDNA, two CD bands are typically observed: a positive λ_max_ at 282 nm (associated with nucleobase stacking) and a negative λ_min_ at 250 nm (related to polynucleotide helicity).^25−27^ The noticeable decrease in absorbance intensity at the positive λ_max_ and negative λ_min_ indicates the influence of f-SWCNTs **1a**–**c** on the dsDNA conformation, including distortion.^27,28^ This effect could be correlated to an increase in dsDNA helix winding angle, accompanied by a decrease in the twist angle.^29−32^ Consequently, it may slightly reduce the number of base pairs per helical turn, leading to a potential perturbation of DNA conformation from B-form to C-form^27,28,33,34^ Among the three f-SWCNTs, **1b** exhibited the most significant reduction in CD intensity for both positive and negative bands. In addition to a 71% reduction in ellipicity, dsDNA **3**@f-SWCNT **1b** (i.e., **5b**) showed a 2.0 nm hypsochromic shift of the positive band and a 2.0 nm bathochromic shift of the negative band compared with the parent dsDNA **3**.

#### Temperature Effect on the Pseudo Duplex-DNA **4** and Pseudo Triplex-DNA **5**

**Figure 6:**
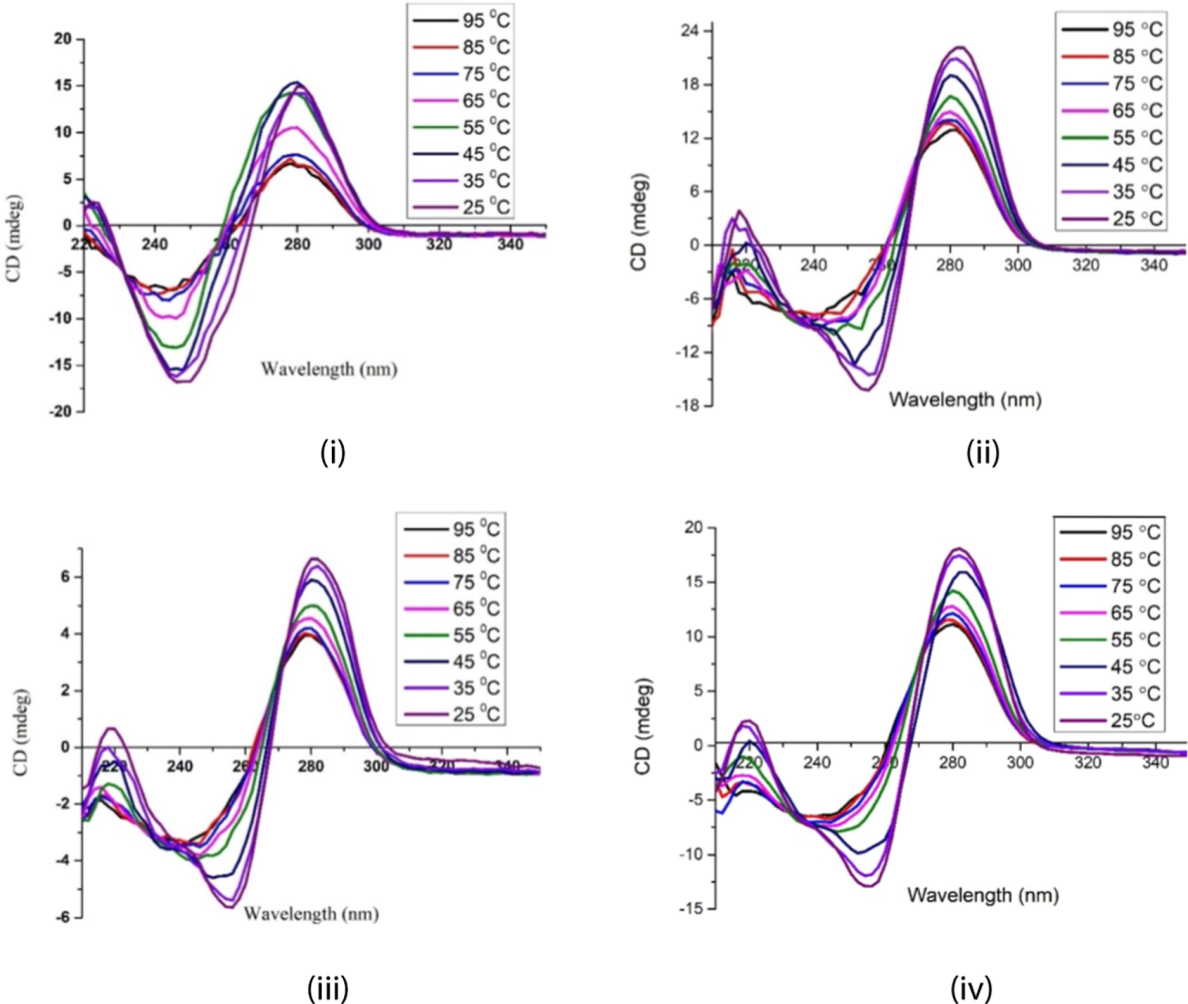
CD spectra of (i) ssDNA **2** alone, (ii) ssDNA **2**@SWCNTs **1a** (i.e., **4a**), (iii) ssDNA **2**@SWCNTs **1b** (i.e., **4b**), and (iv) ssDNA **2**@SWCNTs **1c** (i.e., **4c**) in a buffer solution of pH 7.4 at different temperatures.

Performance of experiments over a range of temperatures, from low to high, would allow the liberation of DNA from the pseudo duplex **4c** and pseudo triplex **5c**. The temperature-dependent CD spectra can not only serve to monitor the denaturation of DNA but also offered insights into the strength of interaction between the SWCNTs and DNA^21,35,36^ We found that the CD intensity of ssDNA **2** diminished with increasing temperature and melted at 65 °C, where the CD intensity significantly decreased (shown by the pink line in Figure 6i).^23,24^ After the complexation of DNA with f-SWCNT **1c** in NaPBS at pH 7.4, the CD intensity of the resultant pseudo duplex **4c** gradually decreased from 25 to 45 °C and then notably dropped at 55 °C (turquoise line in Figure 6iv). Some brown carbon nanotubes **1c** precipitated out of the solution when the temperature exceeded 55 °C, indicating the dehybridization of base pairings between ssDNA **2** and f-SWCNT **1c**. Upon liberation of ssDNA from the hybrid, the resultant ssDNA melted and denatured at 55 °C, which is slightly lower than the melting temperature of the parent ssDNA (65 °C).

In contrast, the temperature-dependent CD spectra characteristic of natural DNA does not apply to the complex of DNA–pristine SWCNT. Dukovic et al.^21^ reported a constant CD intensity for the complex of DNA–pristine SWCNT over the temperature range of 0–50 °C. This stability is attributed to a strongly bound rigid DNA structure formed by π–π stacking between the nucleobases and pristine nanotube sidewall. The pseudo duplex **4c** was regenerated and dissolved in the medium as the temperature was decreased to 55 °C or lower, indicating a reversible process. As a result, the intensity of its CD signatures was restored. The dissociation temperatures for the other two pseudo duplexes **4a** and **4b** were also close to 55 °C, though not as sharp, as shown in Figure 6ii and iii. This dissociation temperature was slightly higher than ambient temperatures but not excessively high to cause damage to many biological materials and systems.

**Figure 7:**
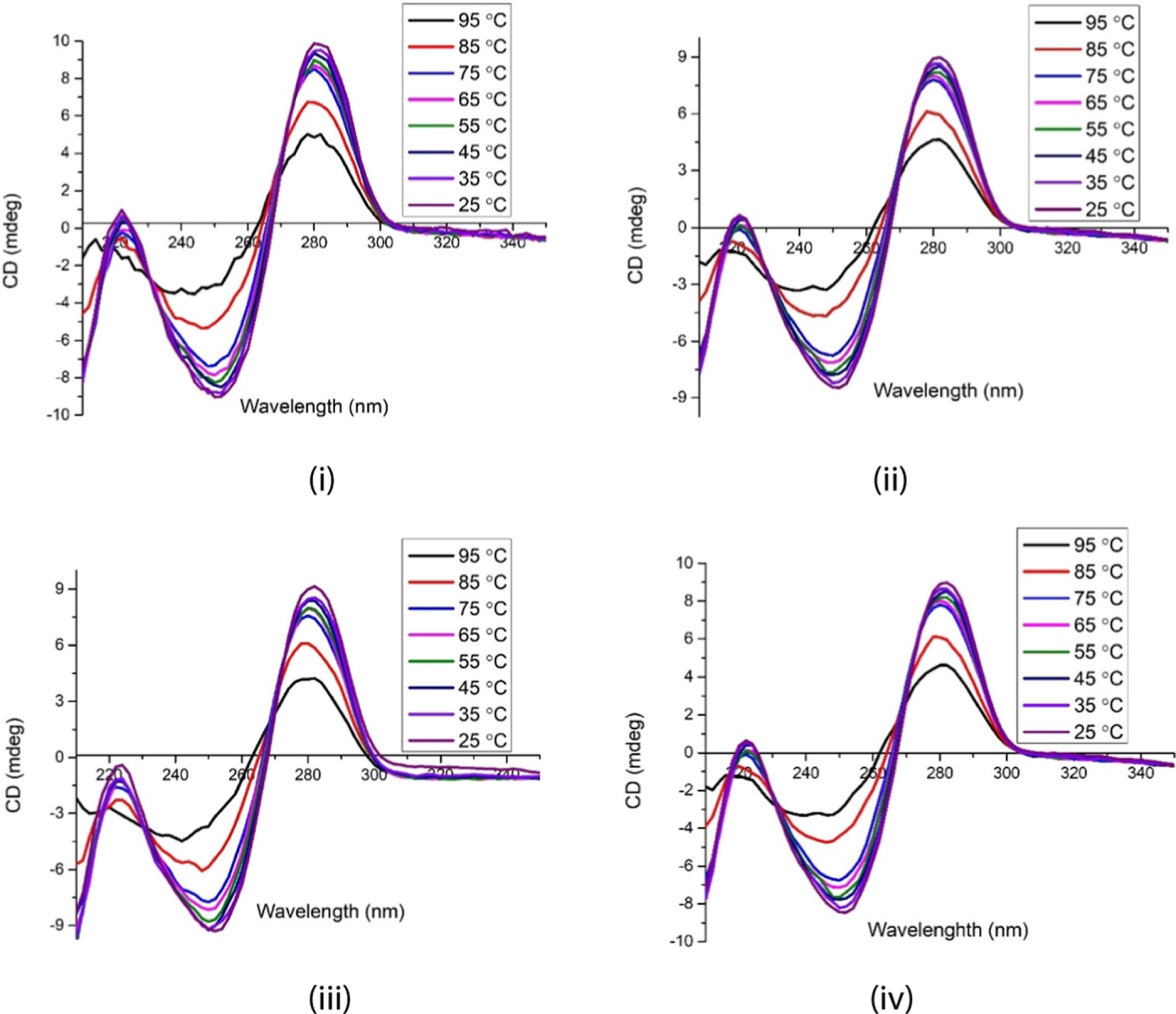
CD spectra of (i) dsDNA **3** alone, (ii) dsDNA **3**@SWCNTs **1a** (i.e., **5a**), (iii) dsDNA **3**@SWCNTs **1b** (i.e., **5b**), and (iv) dsDNA **3**@SWCNTs **1c** (i.e., **5c**) in a buffer solution of pH 7.4 at different temperatures.

The melting of dsDNA involves cleavage of the hydrogen bonds between nucleobase pairs. The intercalative mode of binding often disrupts the hydrogen bonding of dsDNA, leading to variable *T*_m_ values.^37,38^ On the other hand, electrostatic and groove binding interactions with dsDNA may result in indistinct changes in *T*_m_.^39^ CD measurements of dsDNA **3** and pseudo triplex **5** were employed to investigate the bonding strength between DNA and f-SWCNT **1**.^35,40^ As depicted in Figure 7, there was no apparent variation in CD signals from dsDNA **2** and pseudo triplex **5** as the temperature increased from 25 to 90 °C. Denaturation of these structures occurred at 85 °C. Consequently, the binding of f-SWCNTs **1** to dsDNA **3** during the formation of pseudo triplex **5** did not significantly alter the melting behavior of **3** (Figure 7i versus ii, iii, and iv). These findings support the hypothesis that the ligands on f-SWCNTs **1a**–**c** functioned as binders rather than intercalators. They bound to dsDNA **3** through electrostatic or groove-binding interaction, or a combination of both. Furthermore, we observed that f-SWCNT **1a**–**c** started to precipitate out from the buffer solutions containing pseudo triplexes **5a**–**c** at temperature of 85 °C or higher.

#### Microscopic Studies of f-SWCNTs **1a–c**, Hybrid Duplexes **4a–c**, and Hybrid Triplexes **5a–c**

**Figure 8:**
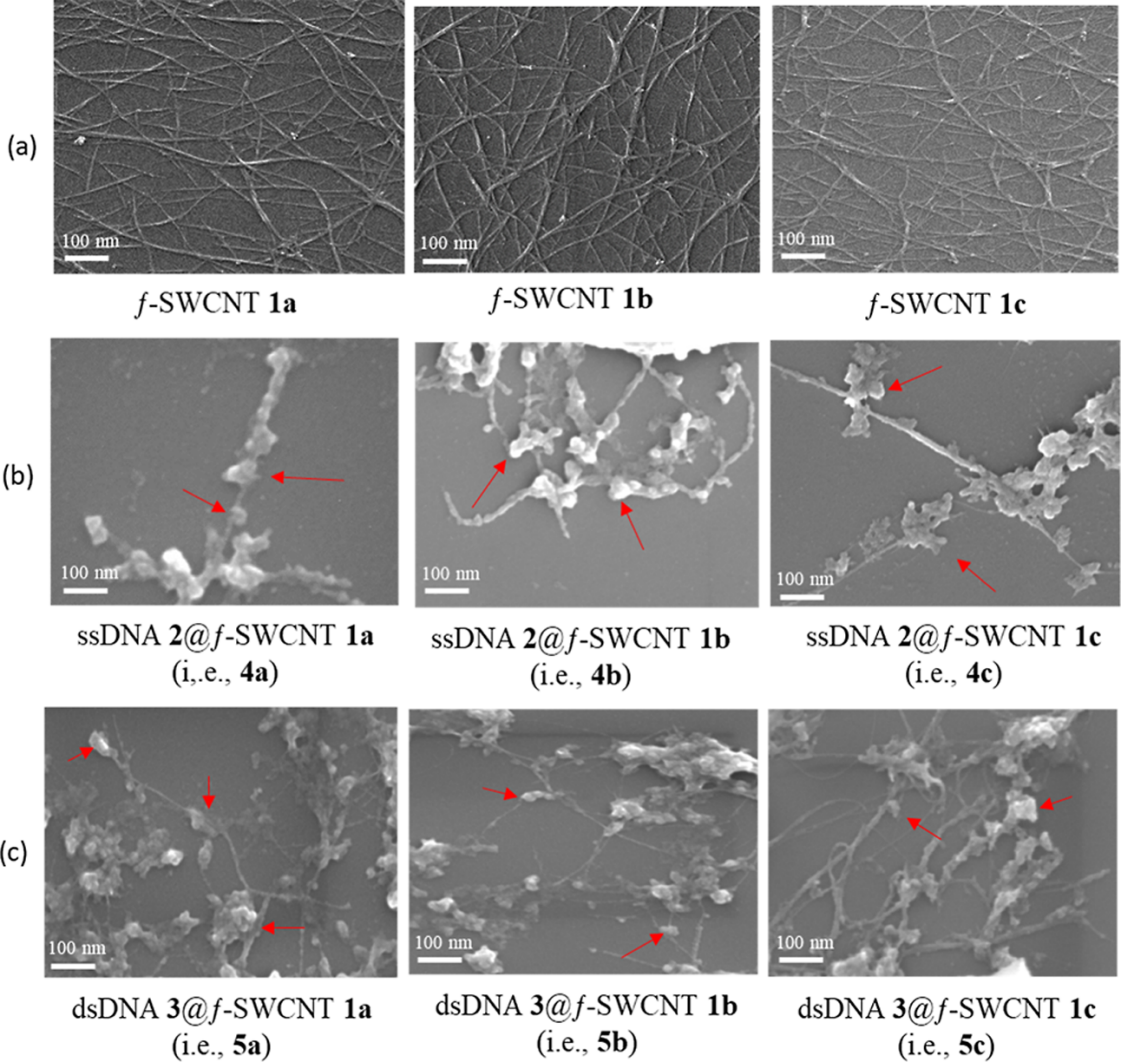
SEM images of f-SWCNTs **1a**–**c** shown in the first row (a), their hybrid duplexes-ssDNA **2**@f-SWCNT **1a**–**c** (i.e., **4a**–**c**) shown in the second row (b), and hybrid triplexes dsDNA **3**@f-SWCNT **1a**–**c** (i.e., **5a**–**c**) in the third row (c). The red arrows indicate the regions where f-SWCNTs **4a**–**c** and f-SWCNTs **5a**–**c** are entwined with ssDNA and dsDNA, respectively.

Direct evidence of hybridization was obtained through the use of scanning electron microscope (SEM), high resolution transmission electron microscope (HRTEM), and atomic force microscope (AFM).^41^ The SEM images in Figure 8 offer insights into the morphology and compositions of f-SWCNTs **1a**–**c** in the first row, hybrid duplexes ssDNA@f-SWCNT **4a**–**c** in the second row, and hybrid triplexes dsDNA@f-SWCNT **5a**–**c** in the third row. The three images in the row (b) related to hybrids **4a**–**c** reveal that f-SWCNTs **1a**–**c** were separately hybridized by ssDNA **2**, appearing as bright white spots on the surface of the tubes, as indicated by the red arrows. This observation provides solid evidence that ssDNA **2** is entwined with f-SWCNTs **1a**–**c**. In the third row (c), SEM images of hybrid triplexes **5a**–**c** show f-SWCNTs **1a**–**c** with partial segments entwined by dsDNA **2**, which appear as bright white spots on the nanotube surfaces, as indicated by the red arrows. These images support the possibility that dsDNA can successfully hybridize with f-SWCNTs **1a**–**c**. Consequently, the designed f-SWCNTs **1a**–**c** can be considered as “artificial” single-stranded DNA.

**Figure 9:**
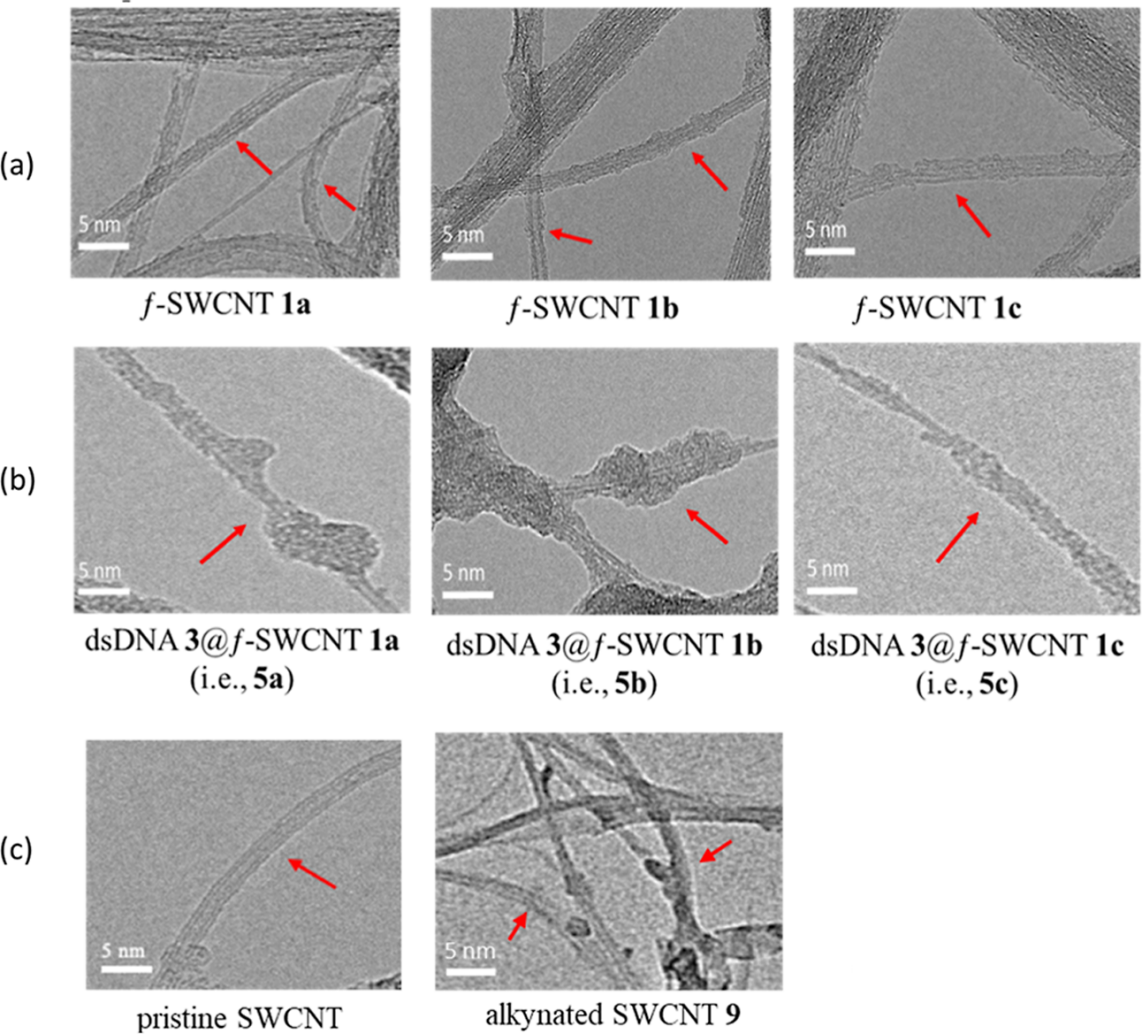
HRTEM images of individual f-SWCNTs **1a**–**c** are shown in the first row (a) and their hybrid triplex-DNAs **5a**–**c** are shown in the second row (b). The pristine SWCNT and alkynated SWCNTs **9**, shown in the third row (c), are provided for comparison. The images of f-SWCNTs were obtained by dispersing them in an aqueous solution and depositing them onto a TEM grid. The red arrows in row (a) indicate the locations of ligand functionalization on SWCNTs, while in row (b), they indicate the regions where f-SWCNTs **1a**–**c** were entwined with dsDNA.

To scrutinize the structural morphology of f-SWCNTs **1a**–**c** and pseudo triplex-DNAs **5a**–**c** at the highest possible resolution, we obtained HRTEM images as depicted in Figure 9. The images in the first row (a) illustrated the grafting of ligands onto the surface of SWCNTs. In the second row (b), transparent tubes entwined by dsDNA on the surface were visible. For comparison, the HRTEM images of pristine SWCNT and alkynated SWCNTs **9** are shown in the third row (c). Previous research by Coleman et al.^42^ shows a complex of dsDNA@pristine SWCNT after hybridization, a process requiring at least 16 days for completion. This intricate formation, facilitated by π–π stacking interactions^43^ could extend up to 35 days.^42^ In contrast, hybridizations between f-SWCNTs **1a**–**c** and dsDNA **3** to form the corresponding triplex-DNAs **5a**–**c** were achieved in just 90 min. This significant reduction in time stands in stark contrast to the prolonged duration associated with the complexation of pristine SWCNT and dsDNA.

The HRTEM images reveal that the diameters of triplex-DNAs **5a**–**c** containing dsDNAs **3**@f-SWCNTs **1a**–**c** were wider compared with those of f-SWCNTs **1a**–**c**, as marked by the red arrows in Figure 9. The surfaces of the triplexes **5a**–**c** appeared much rougher than those of the f-SWCNTs **1a**–**c**, respectively. These microscopy results provide further validation for the distinct morphology between f-SWCNTs **1** and pseudo triplexes **5** (i.e., dsDNAs **3**@f-SWCNTs **1**) and confirm the formation of the triplexes **5** with rod-shaped structures.

**Figure 10:**
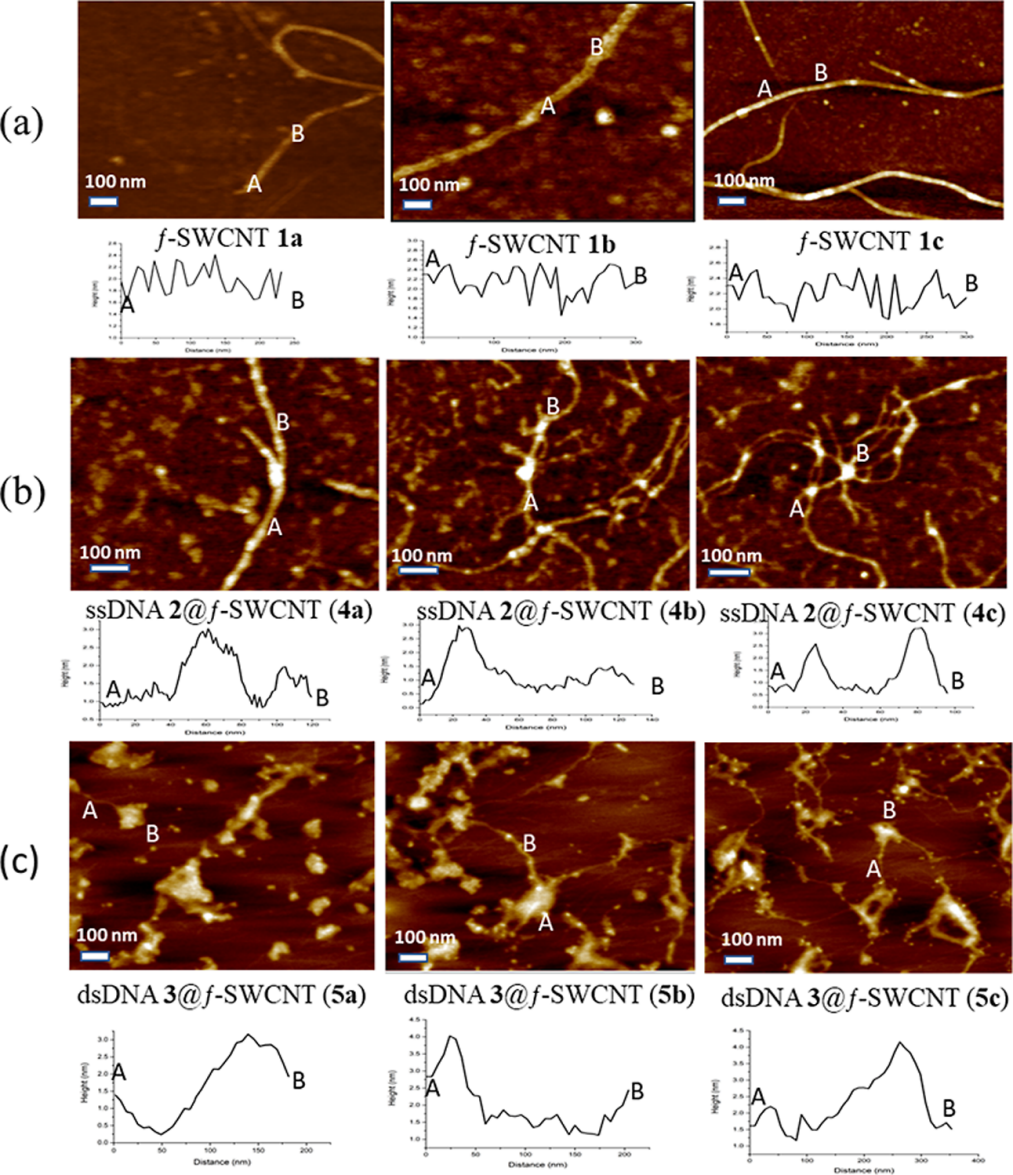
AFM images show bright dot-like structures representing N-HDB-attached ligands, DNAs, or a combination of both. “A” and “B” in each of the nine images represent the start and end points, respectively, of the short f-SWCNTs or their entwinement with DNAs, corresponding to the insets. The inset beneath each image shows the areas selected for the cross-sectional height profiles along the f-SWCNTs. They are (a) individual f-SWCNTs **1a**–**c**, (b) pseudo double-stranded DNAs **4a**–**c** containing ssDNA **2**@f-SWCNTs **1a**–**c**, and (c) pseudo triple-stranded DNAs **5a**–**c** containing dsDNA **3**@f-SWCNTs **1a**–**c**. The peaks in the cross-sectional profiles of (b) and (c) demonstrate the regions where DNAs were entwined.

The AFM analysis played a crucial role on confirmation of the successful hybridization of f-SWCNTs **1a**–**c** with ssDNA **2** and dsDNA **3**. In Figure 10a, three scan size images of f-SWCNTs **1a**–**c** are presented, each accompanied by its cross-sectional profile, as shown in the insets. “A” and “B” mark the corresponding positions in the cross-sectional profiles below. These measurements reveal that the thickness of the tubes ranged from 2.05 to 2.52 nm for the f-SWCNTs with multiple N-HDB-attached ligands. In Figure 10b and c, images of ssDNA **2** and dsDNA **3** entwined with f-SWCNTs **1a**–**c** are shown, respectively. Bright spots on the surfaces of f-SWCNTs represent distinct regions where DNA strands attached. The DNA portions are observed protruding out of the nanotube samples, as evidenced in the cross-sectional profiles. The data on height indicate that ssDNAs **2** and dsDNAs **3** interacted with the N-HDB-attached ligands of f-SWCNTs **1a**–**c**. Peaks in the cross-sectional profiles show regions where DNA strands were bound, while valleys represent areas where ligands were free from hybridization. The heights reached 2.58–3.23 nm in Figure 10b for single-stranded DNA **2** hybridizing with f-SWCNTs **1a**–**c**. Their higher heights of 3.50–4.07 nm are shown in Figure 10c for double-stranded DNA **3** hybridizing with these f-SWCNTs. These data provide strong evidence in support of the successful entwinement of ssDNA **2** and dsDNA **3** with f-SWCNTs **1a**–**c**, forming pseudo duplex-DNA **4** and triplex-DNA **5**, respectively.

Erie et al.^44^ highlighted that a short oligonucleotide might not be long enough to complete a full turn around carbon nanotubes. In contrast, a longer oligonucleotide, such as d(GT)_30_, could make two complete turns and one partial turn around SWCNTs. Thus, it results in three peaks per oligonucleotide in the height profiles. The ability of an oligonucleotide to “wrap” around nanotubes and form a coil depends on the length of the DNA strand.^45,46^ The ssDNA **2** and dsDNA **3** used in our study were 25-mers with lengths of approximately 8.16 nm only. Consequently, it would not be possible for these short DNA strands to coil around the long f-SWCNTs **1**, as shown in our AFM images, SEM, and HRTEM. As a result, we did not observe the uniform periodic peaks in the cross-section profiles of Figure 10c for the formation of triple helical structures, which is characteristic of long DNA strands.

## Conclusions

The development of pristine SWCNTs transformed into f-SWCNTs **1a**–**c**, grafted with multiple N-HDB through amphipathic spacers, represents the successful creation of a class of artificial single-stranded DNA. These f-SWCNTs are designed with a central core of carbon nanotubes having diameters of 0.93–1.77 nm and lengths ranging from 5 to 30 μm. The nanotube surfaces are functionalized with phenoxy–triazole–(ethylene glycol)–spacers; each spacer is a string with approximately 50 carbons. At the end of each spacer, an N-HDB is attached, with variations like imidazolidin-2-one, hydantoin, or urazole. These N-HDB moieties are strategically designed with one to three binding sites to interact with the nucleobases of DNA.

The entwinement of dsDNAs and f-SWCNTs **1a**–**c** through nucleobases and N-HDB results in the formation of pseudo triplex-DNA. This groundbreaking phenomenon marks the premier instance where f-SWCNTs with multiple N-HDB hybridize with dsDNA, functioning as groove binders to form triple-stranded DNAs. These pseudo triplexes can dissociate at 85 °C and regenerate their hybrid nanostructures upon temperature reduction. Additionally, ssDNA can hybridize with f-SWCNTs **1a**–**c** to form pseudo duplex-DNA through Watson–Crick type hydrogen bonding. The melting of ssDNA **2**@f-SWCNTs **1a**–**c** (i.e., **4a**–**c**) occurs close to 55 °C, a temperature 30 °C lower than that of the normally complementary dsDNA **3** (85 °C).

These grafted SWCNTs **1a**–**c**, with enhanced dispersibility in aqueous media and reduced toxicity demonstrate significant potential for applications in biotechnology and biomedicine.^47^ As new SWCNTs with ligands capable of special binding, these nanotubes can serve as targeted drug carriers and ensure the efficient delivery of chemotherapeutics to specific cells or tissues while minimizing off-target effects.^48^ Their use also provides an additional method for controlled anticancer drug release. Furthermore, their ability to interact with nucleic acids makes them suitable for gene delivery and facilitates effective binding and protection of DNA and RNA.^49^ Meanwhile, their nanoscale dimensions enable cellular uptake via endocytosis, which is particularly relevant for therapeutic approaches in gene editing or silencing.

## Data Availability

The data underlying this study are available in the published article and itsSupporting Information.

## Supporting Information Available

General procedures for the synthesis of compounds **8a**–**c** and f-SWCNTs **1a**–**c**, along with their spectroscopic data; hybridization of ssDNA and dsDNA with f-SWCNTs **1a**–**c**; characterization of f-SWCNTs **1a**–**c** by use of IR, Raman, and circular dichroism spectroscopy, as well as thermogravimetric analysis; references; spectra (PDF) (PDF)
