# Supplementary material for: Biochemical Nanotubes Containing Heterocycles as Artificial Strands for Pseudo Duplex and Triplex DNA Formation
Source: J Phys Chem B. 2025 Mar 11;129(11):2903–14. doi: 10.1021/acs.jpcb.4c08079 (PMC11931538; doi:10.1021/acs.jpcb.4c08079)
Supplement: Supplementary file 1 — jp4c08079_si_001.pdf [file jp4c08079_si_001.pdf]

# Supporting Information

# Biochemical Nanotubes Containing Heterocycles as Artificial Strands for Pseudo Duplex and Triplex DNA Formation

*Jih Ru Hwu,[†,‡,]* Deepa Rohidas Landge,[†,‡] Wen-Chieh Huang,[†,‡] Jia-Cherng Horng,[†,‡] Yu-Chen Hu,[‡,§] Kuo Chu Hwang,[†,‡] Chun-Cheng Lin,[†,‡] and Shwu-Chen Tsay[†,‡,]**

[†]Department of Chemistry, National Tsing Hua University, Hsinchu 30044, Taiwan

[‡]Frontier Research Center on Fundamental and Applied Sciences of Matters, National Tsing Hua University, Hsinchu 300044, Taiwan

[§]Department of Chemical Engineering, National Tsing Hua University, Hsinchu 300044, Taiwan

Email: jrhwu@mx.nthu.edu.tw; sctsay@mx.nthu.edu.tw

**General Procedure.** All reactions were carried out in oven-dried glassware (120 °C) under an atmosphere of nitrogen unless as indicated otherwise. Ethyl acetate (EtOAc) was dried and distilled from $CaH_2$; THF was dried by distillation from sodium and benzophenone under an atmosphere of nitrogen. Acetic acid (AcOH), dichloromethane ($CH_2Cl_2$), ethyl acetate, hexanes, methanol (MeOH), and tetrahydrofuran (THF) were purchased from Mallinckrodt Chemical Co. Chemicals purchased from Tokyo Chemical Industry Co. Ltd. were imidazolidin-2-one and urazole. Copper iodide (CuI), hydantoin, pentaethylene glycol, and sodium hydride were purchased from Aldrich Chemical Co. Hydrochloric acid, potassium carbonate, sodium carbonate, and sodium hydroxide were purchased from Showa Chemical Co. 1,8-Diazabicycloundec-7-ene (DBU) was purchased from Wolfen Co. Dimethylformamide (DMF) and triethylamine were

purchased from Tedia Company Inc. Isoamyl nitrite, 4-nitrophenol, and *p*-toluenesulfonyl chloride were purchased from Acros Organics. *N*-Methyl-2-pyrrolidinone (NMP) was purchased from ECHO Chemical Co., Ltd. Single-wall carbon nanotube (SWCNT) was purchased from Chengdu Organic Chemicals Co. Ltd. (Trimethylsilyl)trifluoromethane sulfonate was purchased from Merck Inc.

Analytical thin layer chromatography (TLC) was performed on precoated plates (silica gel 60, F-254) purchased from Merck Inc. Infrared (IR) spectra were measured on Jasco spectrometer FT−IR 4200. Absorption intensities are recorded by the following abbreviations: s, strong; m, medium; w, weak. Proton NMR spectra were obtained on a Varian Mercury-400 (400 MHz), Bruker-DM-600, or Varian 500 spectrometer by use of chloroform-*d* ($CDCl_3$) as the solvent. Their chemical shifts were referenced to the residual protonated solvent ($\delta$ 7.24 for chloroform). Carbon-13 NMR spectra were obtained on a Varian Mercury-400 (100 MHz), Bruker-DM-600 (150MHz) HR, or Varian 500 (125 MHz) spectrometer by use of chloroform-*d* ($CDCl_3$) as the solvent. Their chemical shifts are referenced to the center of the $CDCl_3$ triplet ($\delta$ 77.0 ppm). Multiplicities are recorded by the following abbreviations: s, singlet; d, doublet; t, triplet; q, quartet; m, multiplet; *J*, coupling constant (hertz). High-resolution mass spectra (HRMS) were obtained by means of a JEOL JMS-HX110 mass spectrometer. The Raman spectra were recorded by use of a LabRAM HR high-resolution Jobin YvonHoriba HR 800 Raman spectrometer by use of the 532 nm line of a He–Ne laser as excitation source.

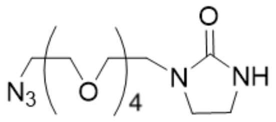

**8a**

**1-(14-Azido-3,6,9,12-tetraoxatetradecyl)imidazolidin-2-one (8a).** To a solution containing imidazolidin-2-one **6a**[1] (0.263 g, 3.06 mmol, 3.0 equiv) in DMF (4.3 mL) was added NaH (60% in mineral oil, 41.0 mg, 1.02 mmol, 1.0 equiv). After the reaction mixture was stirred at room temperature for 2.0 h, 14-azido-3,6,9,12-tetraoxatetradecyl 4-methylbenzenesulfonate **7**[2] (0.426 g, 1.02 mmol, 1.0 equiv) was added to the mixture. The reaction mixture was stirred at room temperature for another 18 h and then extracted with EtOAc (4 × 5.0 mL). The combined organic layers were washed with brine (5.0 mL), dried over $MgSO_4$ (s), filtered, and concentrated under reduced pressure. The residue was purified by use of gravity column chromatography on silica gel (2.0% MeOH in $CH_2Cl_2$ as the eluent) to give the azide **8a** (0.246 g, 0.744 mmol) in 73% yield as a yellowish oil: $^1$H NMR ($CDCl_3$, 400 MHz) $\delta$ 3.74–3.60 (m, 16 H, 8 × $CH_2O$), 3.59–3.54 (m, 2 H, $CH_2N$), 3.47–3.35 (m, 6 H, $NCH_2CH_2N$ + $CH_2N_3$); $^{13}$C NMR ($CDCl_3$, 100 MHz) $\delta$ 162.66 (C=O), 71.03, 70.37, 70.31, 70.26, 70.13, 69.94, 69.79, 69.73, 50.42 ($CH_2N_3$), 46.18, 43.15, 38.18; IR (KBr) 3363 (br, NH), 2920 (s), 2106 (s, $N_3$), 1694 (s, C=O), 1452 (m), 1350 (w), 1276 (s), 1103 (s) cm$^{-1}$; HRMS (ESI-TOF) *m/z* [M + Na]$^+$ calcd for $C_{13}H_{25}N_5O_5$ + Na 354.1753, found 354.1762.

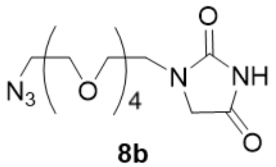

**8b**

**1-(14-Azido-3,6,9,12-tetraoxatetradecyl)imidazolidine-2,4-dione (8b).** To a solution containing imidazolidine-2,4-dione **6b**[3] (0.299 g, 2.99 mmol, 3.0 equiv) in DMF (2.0 mL) was added $K_2CO_3$ (0.483 g, 3.49 mmol, 3.5 equiv). After the reaction mixture was stirred at 50 °C for

90 min, 14-azido-3,6,9,12-tetraoxatetradecyl 4-methylbenzenesulfonate **7**[2] (0.417 g, 0.998 mmol, 1.0 equiv) in DMF (2.0 mL) was added to the mixture. The reaction mixture was stirred at 110 °C for 24 h and then extracted with EtOAc (3 × 7.0 mL). The combined organic layers were washed with brine (5.0 mL), dried over $MgSO_4$ (s), filtered, and concentrated under reduced pressure. The residue was purified by use of gravity column chromatography on silica gel (90% EtOAc in hexane as the eluent) to give the azide **8b** (0.307 g, 0.889 mmol) in 89% yield as a colourless oil: $^1$H NMR ($CDCl_3$, 400 MHz) $\delta$ 3.96 (s, 2 H, $NCH_2CO$), 3.72–3.60 (m, 16 H, 8 × $CH_2O$), 3.59–3.56 (m, 2 H, $CH_2N$), 3.38 (t, $J$ = 5.04 Hz, 2 H, $CH_2N_3$); $^{13}$C NMR ($CDCl_3$, 100 MHz) $\delta$ 171.25 (C=O), 157.93 (C=O), 70.64, 70.62, 70.53, 70.51, 70.46, 70.04, 69.94, 67.23, 50.69 ($CH_2N_3$), 46.46 ($CH_2NCO$), 37.95 ($NCH_2CO$); IR (KBr) 3285 (br, NH), 2925 (s), 2104 (m, $N_3$), 1769 (w, C=O), 1709 (s, C=O), 1615 (m), 1458 (m), 1234 (w), 1100 (m) cm$^{-1}$; HRMS (ESI-TOF) $m/z$ $[M + Na]^+$ calcd for $C_{13}H_{23}N_5O_6$ + Na 368.1546, found 368.1551.

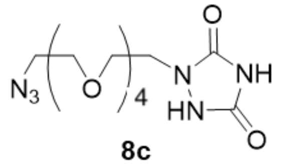


**8c**

**1-(14-azido-3,6,9,12-tetraoxatetradecyl)-1,2,4-triazolidine-3,5-dione (8c).** To a solution containing 1,2,4-triazolidine-3,5-dione **6c**[4] (0.302 g, 2.99 mmol, 3.0 equiv) in DMF (5.3 mL) was added NaH (60% in mineral oil, 40.5 mg, 0.998 mmol, 1.0 equiv). After the reaction mixture was stirred at room temperature for 2.0 h, 14-azido-3,6,9,12-tetraoxatetradecyl 4-methylbenzenesulfonate **7**[2] (0.417 g, 0.998 mmol, 1.0 equiv) was added to the mixture. The reaction mixture was stirred at 110 °C for 20 h and then extracted with EtOAc (4 × 5.0 mL). The combined organic layers were washed with brine (6.0 mL), dried over $MgSO_4$ (s), filtered, and concentrated under reduced pressure. The residue was purified by use of gravity column

chromatography on silica gel (5.0% MeOH in $CH_2Cl_2$ as the eluent) to give the azide **8c** (0.280 g, 0.809 mmol) in 81% yield as a yellowish oil: $^1$H NMR ($CDCl_3$,400 MHz) $\delta$ 3.72–3.67 (m, 16 H, 8 × $CH_2O$), 3.66–3.62 (m, 2 H, $CH_2N$), 3.39 (t, $J$ = 5.02 Hz, 2 H, $CH_2N_3$); $^{13}$C NMR ($CDCl_3$, 125 MHz) $\delta$ 153.51 (C=O), 153.36 (C=O), 70.59, 70.57, 70.37, 70.34, 70.13, 70.03, 69.99, 69.91, 50.63 ($CH_2N_3$), 45.40 ($CH_2NCO$); IR (KBr) 3500 (br, NH), 2923 (s), 2107 (s, $N_3$), 1714 (s, C=O), 1621 (s, C=O), 1512 (m), 1455 (w), 1115 (m) cm$^{-1}$; HRMS (ESI-TOF) *m/z* $[M + H]^+$ calcd for $C_{12}H_{22}N_6O_6 + H^+$ 347.1695, found 347.1679.

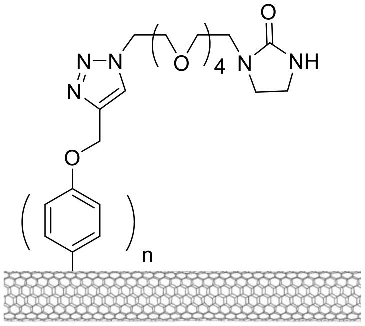


**1a**

**(Poly imidazolidine-2-one)-SWCNT (1a).** Alkynated SWCNT **9**[5] (31.7 mg, 1.01 mmol of carbon) was dispersed in DMF by sonication at room temperature for 10 min. To this black solution was added 1-(14-azido-3,6,9,12-tetraoxatetradecyl)imidazolidin-2-one **8a** (0.331 mg, 1.01 mmol, 1.05 equiv), CuI (1.05 g, 5.53 mmol, 5.5 equiv), and DBU (2.98 g, 14.1 mmol, 14 equiv). After the reaction mixture was stirred at 110 °C for 96 h, the resultant paste was cooled down to room temperature, diluted with THF (3 × 10 mL) and then filtered through a poly(tetrafluoroethene) membrane (450 nm pore size). The collected solids were washed with DMF (5 × 10 mL) followed by THF (3 × 10 mL). Subsequently, the solids were dispersed in *N*-methyl-2-pyrrolidinone (NMP, 2.0 mL) by sonication for 10 min. After the solution stood for 2–3 min, the less dispersible alkynated SWCNT **9** settled down. The supernatant was taken and carefully filtered through a poly(tetrafluoroethene) membrane. The solids on the membrane were collected, which were then

washed with $CH_2Cl_2$ (3 × 10 mL) to remove NMP. The remaining solids were dried at 40 °C to give the material SWCNT **1a** (40.5 mg) as dark brown solids: IR (KBr) 3299 (br, NH), 2923 (m), 1685 (s, C=O), 1492 (m), 1449 (m), 1349 (w, C-N), 1273 (s), 1100 (m) $cm^{-1}$.

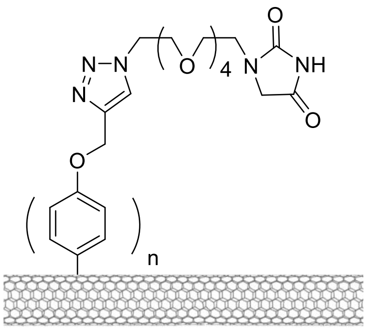


**1b**

**(Poly imidazolidine-2,4-dione)-SWCNT (1b).** Alkynated SWCNT **9**[5] (31.7 mg, 1.02 mmol of carbon) was dispersed in DMF (10.2 mL) by sonication at room temperature for 10 min. To this black solution was added azide **8b** (0.346 g, 1.01 mmol, 1.05 equiv), CuI (1.05 g, 5.50 mmol, 5.5 equiv), and DBU (2.98 g, 14.1 mmol, 14 equiv). After the reaction mixture was stirred at 110 °C for 96 h, the resultant paste was cooled down to room temperature, diluted with THF (5 × 10 mL) and then filtered through a poly(tetrafluoroethene) membrane (450 nm pore size). The collected solids were washed with DMF (5 × 10 mL) followed by THF (5 × 10 mL). Subsequently, the solids were dispersed in NMP (2.0 mL) by sonication for 10 min. After the solution stood for 2–3 min, the less dispersible alkynated SWCNT **9** settled down. The supernatant was taken and carefully filtered through a poly(tetrafluoroethene) membrane. The solids on the membrane were collected, which were then washed with $CH_2Cl_2$ (4 × 10 mL) to remove NMP. The remaining solids were dried at 40 °C to give the material SWCNT **1b** (41.2 mg) as dark brown to black solids: IR (KBr) 3408 (br, NH), 2924 (s), 2854 (m), 1728 (m, C=O), 1632 (w, C=O), 1463 (m), 1374 (w, C-N), 1260 (w), 1166 (w), $cm^{-1}$.

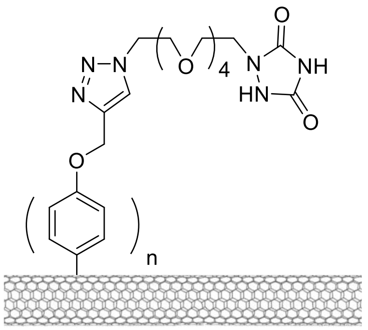

**1c**

**(Poly triazolidine-3,5-dione)-SWCNT (1c).** Alkynated SWCNT **9**[5] (31.7 mg, 1.01 mmol of carbon) was dispersed in DMF (6.0 mL) by sonication at room temperature for 20 min. To this black solution was added azide **8c** (0.357 g, 1.03 mmol, 1.05 equiv), CuI (1.07 g, 5.66 mmol, 5.5 equiv), and DBU (2.19 g, 14.1 mmol, 14 equiv). After the reaction mixture was stirred at 110 °C for 96 h, the resultant paste was cooled down to room temperature, diluted with THF (3 × 10 mL) and then filtered through a poly(tetrafluoroethene) membrane (450 nm pore size). The collected solids were washed with DMF (5 × 10 mL) followed by THF (3 × 10 mL). The solids were then dispersed in NMP (2.0 mL) by sonication for 10 min. After the solution was left to stand for 2–3 min, the less dispersible alkynated SWCNT **4** settled down. The supernatant was taken and carefully filtered through a poly(tetrafluoroethene) membrane. The solids on the membrane were collected, which were then washed with $CH_2Cl_2$ (3 × 10 mL) to remove NMP. The remaining solids were dried at 40 °C to give the material SWCNT **1c** (41.6 mg) as black solids: IR (KBr) 3297 (br, NH), 2993 (m), 1797 (s, C=O), 1746 (m, C=O), 1626 (m), 1337 (w, C-N), 1288 (m), 1219 (s), 1093 (s) $cm^{-1}$.

**Hybridization of Single-stranded DNA (ssDNA) or Double-stranded (dsDNA), respectively, with *f*-SWCNTs 1a–c**. In a series of experiments, three separate sets of sodium phosphate-buffered saline (50 mM NaPBS, 0.501 mL) containing an excess of SWCNTs labeled as **1a** (1.05 mg), **1b** (1.01 mg), or **1c** (1.02 mg) were added to buffers that contained ssDNA or dsDNA (0.480

mg) at a concentration of 0.502 mM. These mixtures were then subjected to bath sonication at 0 °C with a 35 kHz bath sonicator (Sonics, VX130 PB, 3W) for a duration of 90 minutes.[6] The resultants were centrifuged at 13,000 rpm (Beckman GS-15R) for 30 minutes to remove insoluble functionalized SWCNTs. The supernatants obtained after centrifugation were stored at –20 °C until further use.

**Characterization of *f*-SWCNTs 1a–c by IR and Raman spectroscopy.** To identify the functional groups on the grafted SWCNTs **1a**–**c**, attenuated total reflection FT-IR (ATR-FTIR) spectrometer was used. The surface of ATR was cleaned by methanol.[7] Each of the SWCNTs **1a** (10.1 mg), **1b** (15.2 mg), and **1c** (50.1 mg) were covered with the infrared reflecting medium and fixed on ATR-FTIR. The frequency range of FTIR spectrum was set from 4000 to 400 $cm^{-1}$. All of the new SWCNTs were fully characterized and SWCNT **1c** was described as a representative example. Its IR spectrum exhibited a broad absorption band at 3297 $cm^{-1}$ for the N–H stretching vibration and a medium (m) band at 2993 $cm^{-1}$ for the =C–H stretching vibration. Other characteristic peaks included a strong band at 1797 $cm^{-1}$ and a medium band at 1746 $cm^{-1}$, which were associated with the imide C=O groups. A weak band at 1337 (w) $cm^{-1}$ was associated with the triazole C–N stretching vibration.

The SWCNTs **1a** (3.01 mg/mL), **1b** (3.02 mg/mL), **1c** (3.02 mg/mL), alkynated SWCNT **9** (3.01 mg/mL), and pristine SWCNT (3.01 mg/mL) were dispersed in water. Each sample (50.0 μL) was deposited on a $Si/SiO_2$ substrate and dried at room temperature. After drying, the samples were analysed by use of the LabRAM HR high resolution Jobin YvonHoriba HR 800 Raman spectrometer with 532 nm He-Ne laser as excitation source. Additionally, excitation of the samples **1a**, **1b**, and **1c** at 532 nm by Raman instrument generated humps at 1341, 1344, and 1342 $cm^{-1}$, respectively, as shown in the main text of Figures 3(iii)–(v). They were attributed to $sp^3$-

hybridized carbons in the hexagonal framework.[8] Nevertheless, these D-band absorptions were not observed in the Raman spectrum of the pristine SWCNT in Figure 3(i). It exhibited only a G-band at about 1589 $cm^{-1}$, which is associated with $sp^2$-hybridized carbons. Moreover, the radial breathing mode (RBM) frequencies in Figure 3 were used to calculate the diameters ($d_t$) of *f*-SWCNTs **1**. According to the RBM frequency $\omega_{RBM}$ ($cm^{-1}$) = A/$d_t$ (nm) + B, where A = 234 $cm^{-1}$ and B = 10 $cm^{-1}$, the SWCNT diameters were calculated as shown in Table 1 of the main text.[9]

**Thermogravimetric Analysis (TGA) of *f*-SWCNTs 1a–c.** Thermogravimetric analyses were performed with a TGA Q500 V20.13 instrument.[5,6] Samples were analysed in platinum pans at a heating rate of 10 °C/min from ambient temperature to 900 °C in an atmosphere of $N_2$. Sample masses of *f*-SWCNTs **1a** (2.02 mg), **1b** (2.01 mg), **1c** (2.02 mg), and alkynated SWCNT **9** (2.01 mg) were used. Their functionalization degree was estimated by use of Campidelli's method[5,6] with an average of one (phenoxy)alkynyl ligand per 49, 47, 48, and 41 carbon atoms, respectively. The calculation details are provided in Figure S1.

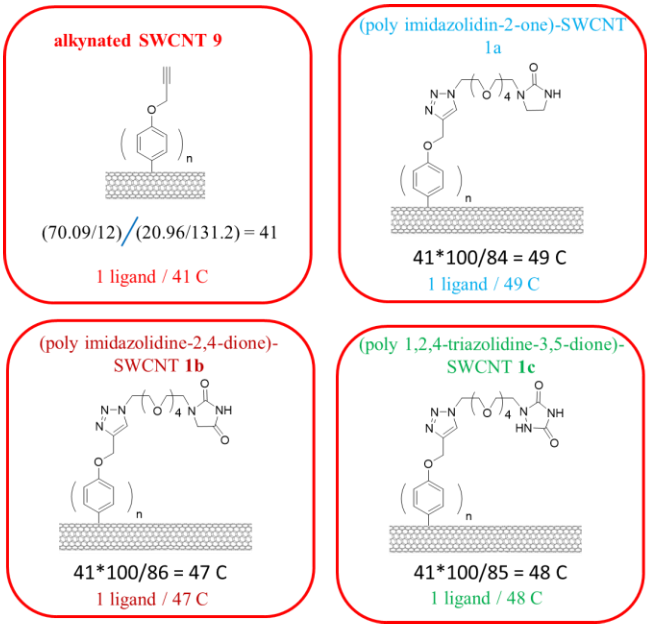


**Figure S1.** TGA calculations for determination of the average number of carbon atoms and the ratio of ligands per carbon atom for the (phenoxy)alkynyl ligand and *f*-SWCNTs 1**a**–**c**, respectively.

**Circular Dichroism (CD) spectroscopy of *f*-SWCNTs 1a–c.** The CD spectra of *f*-SWCNTs **1a**–**c** were recorded on a CD spectrometer Model 410 (AVIV Biomedical Inc.). Each sample (1.0 mg) of **1a**, **1b**, and **1c** was dispersed into a NaPBS buffer solution (0.500 mL) and sonicated at room temperature for 15 min. Their CD spectra are shown in Figure S2.

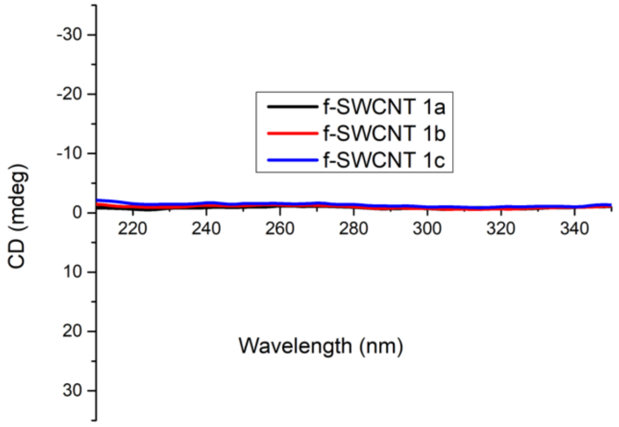


**Figure S2.** CD spectra of *f*-SWCNTs **1a**–**c** in a NaPBS buffer solution at 25 °C.

## References


1. Cai, L.; Qu, B.; Hurtle, B. T.; Dadiboyena, S.; Diaz-Arrastia, R.; Pike, V. W. Candidate PET radioligand development for neurofibrillary tangles: two distinct radioligand binding sites identified in postmortem Alzheimer's disease brain. *ACS Chem. Neurosci.* **2016**, *7*, 897–911.
2. Ai, T.; Qiu, L.; Xie, J.; Geraghty, R. J.; Chen, L. Design and synthesis of an activity-based protein profiling probe derived from cinnamic hydroxamic acid. *Bioorg. Med. Chem.* **2016**, *24*, 686–692.
3. Shintani, Y.; Kato, K.; Kawami, M.; Takano, M.; Kumamoto, T. Direct N1-selective alkylation of hydantoins using potassium bases. *Chem. Pharm. Bull.* **2021**, *69*, 407–410.
4. Kolb, V. M.; Dworkin, J. P.; Miller, S. L. Alternative bases in the RNA world: the prebiotic synthesis of urazole and its ribosides. *J. Mol. Evol.* **1994**, *38*, 549–557.
5. Campidelli, S.; Ballesteros, B.; Filoramo, A.; Díaz, D. D.; de la Torre, G.; Torres, T.; Rahman, G. M. A.; Ehli, C.; Kiessling, D.; Werner, F. et al. Facile decoration of

functionalized single-wall carbon nanotubes with phthalocyanines via “click chemistry”. *J. Am. Chem. Soc.* **2008**, *130*, 11503–11509.

6. Hwu, J. R.; Kapoor, M.; Li, R.-Y.; Lin, Y.-C.; Horng, J.-C.; Tsay, S.-C. Synthesis of Nucleobase-Functionalized Carbon Nanotubes and Their Hybridization with Single-Stranded DNA. *Chem. Asian J.* **2014**, *9*, 3408–3412.

7. Hsieh, Y.-C.; Chou, Y.-C.; Lin, C.-P.; Hsieh, T.-F.; Shu, C.-M. Thermal analysis of multi-walled carbon nanotubes by Kissinger's corrected kinetic equation. *Aerosol Air Qual. Res.* **2010**, *10*, 212–218.

8. Pimenta, M. A.; Dresselhaus, G.; Dresselhaus, M. S.; Cançado, L. G.; Jorio, A.; Saito, R. Studying disorder in graphite-based systems by Raman spectroscopy. *Phys. Chem. Chem. Phys.* **2007**, *9*, 1276–1290.

9. Ö berg, S.; Adjizian, J.-J.; Erbahar, D.; Rio, J.; Humbert, B.; Dossot, M.; Soldatov, A.; Lefrant, S.; Mevellec, J.-Y.; Briddon, P. et al. Effect of functionalization and charging on resonance energy and radial breathing modes of metallic carbon nanotubes. *Phys. Rev. B* **2016**, *93*, 045408.

# Spectral Data of New Compounds

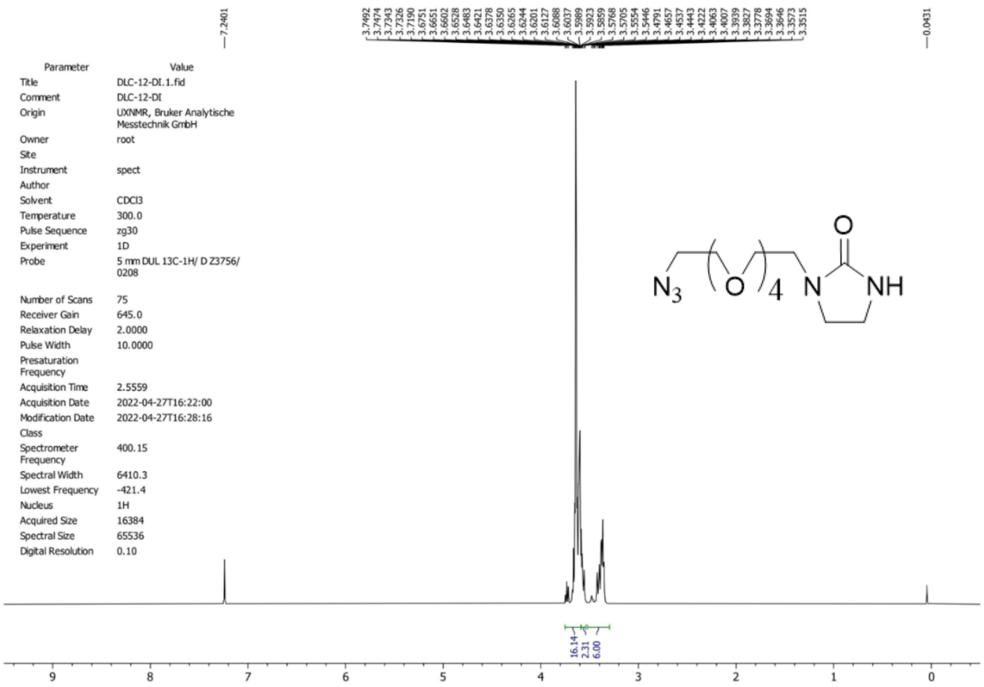

$^1$H NMR spectrum of compound **8a**

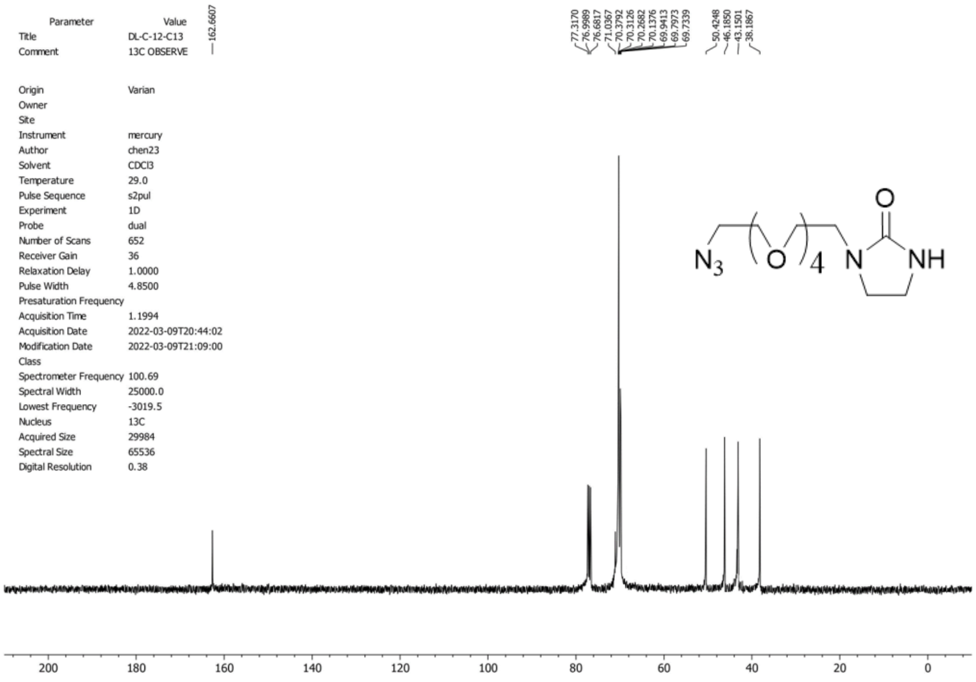

$^{13}$C NMR spectrum of compound **8a**

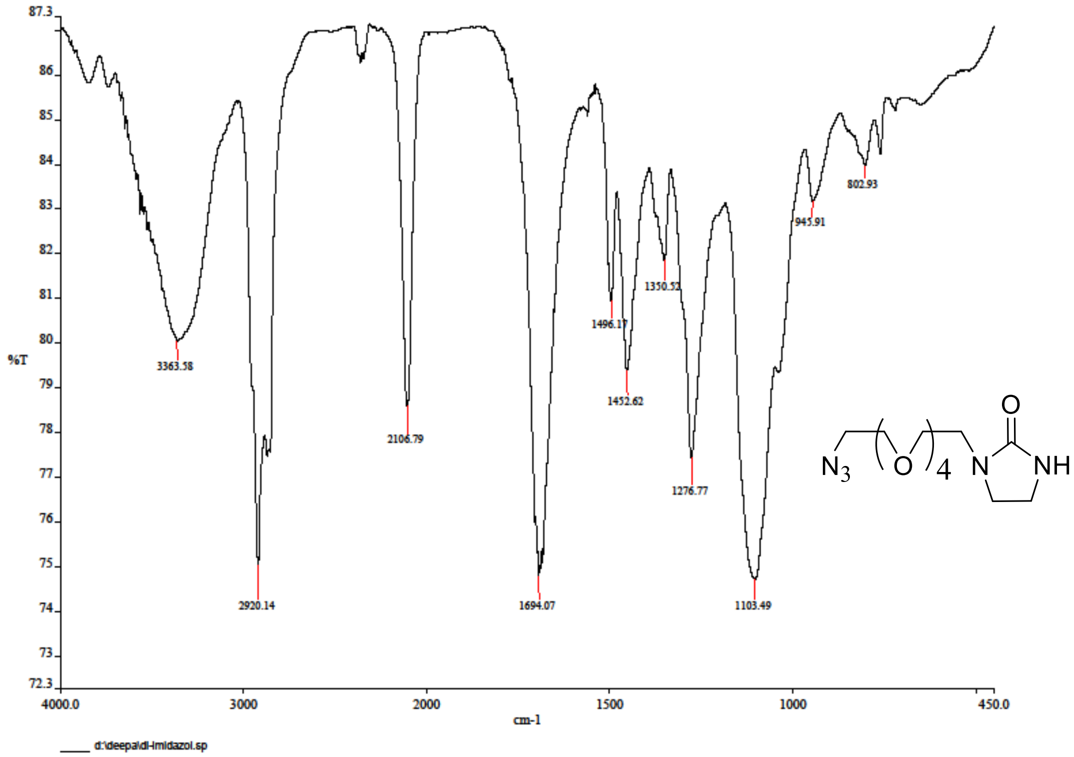


IR spectrum of compound **8a**

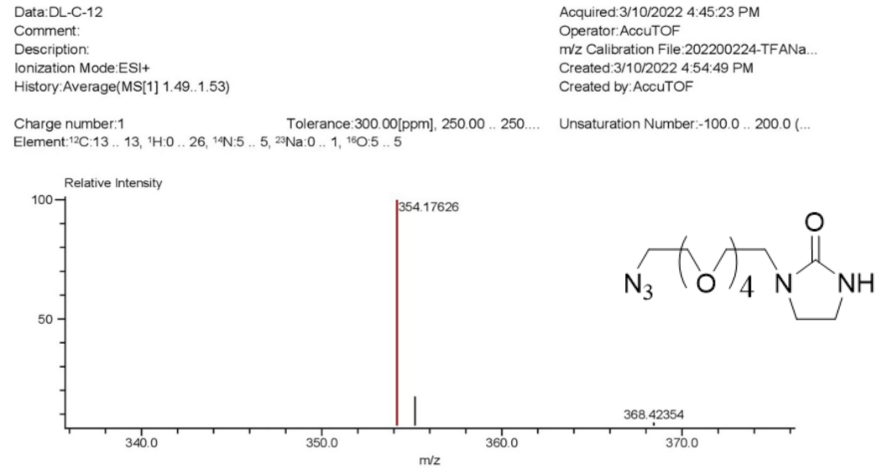


| Mass | Intensity | Calc. Mass | Mass Difference [mDa] | Mass Difference [ppm] | Possible Formula |
|---|---|---|---|---|---|
| 354.17626 | 16373.68 | 354.17534 | 0.92 | 2.59 | $^{12}C_{13}{}^{1}H_{25}{}^{14}N_{5}{}^{23}Na_{1}{}^{16}O_{5}$ |

Mass spectrum of compound **8a**

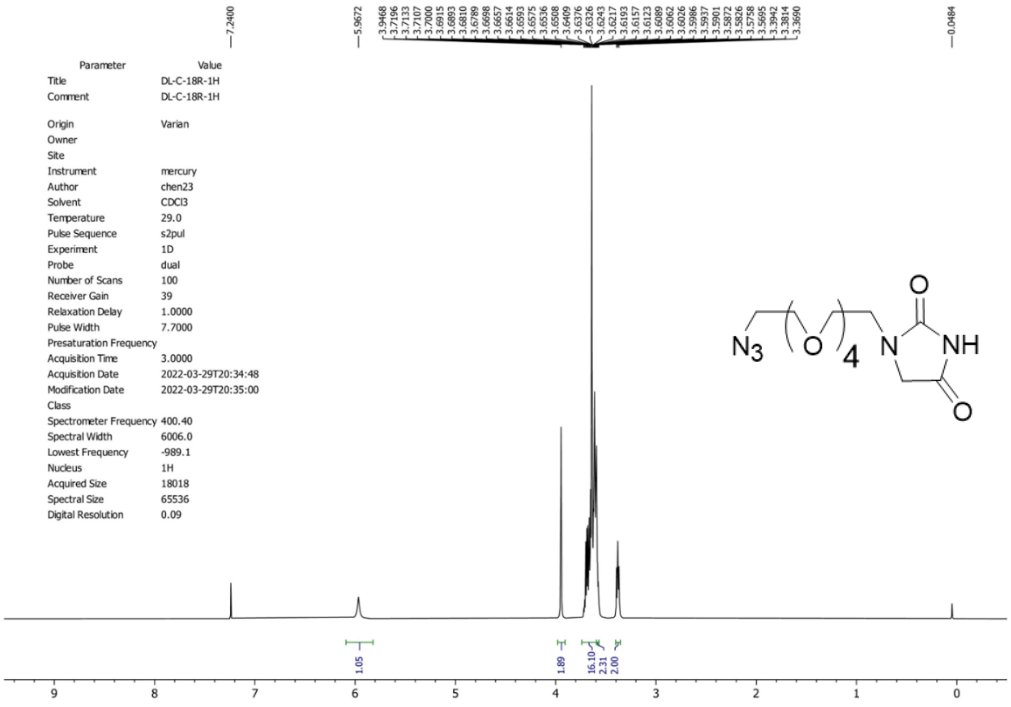

$^{1}$H NMR spectrum of compound **8b**

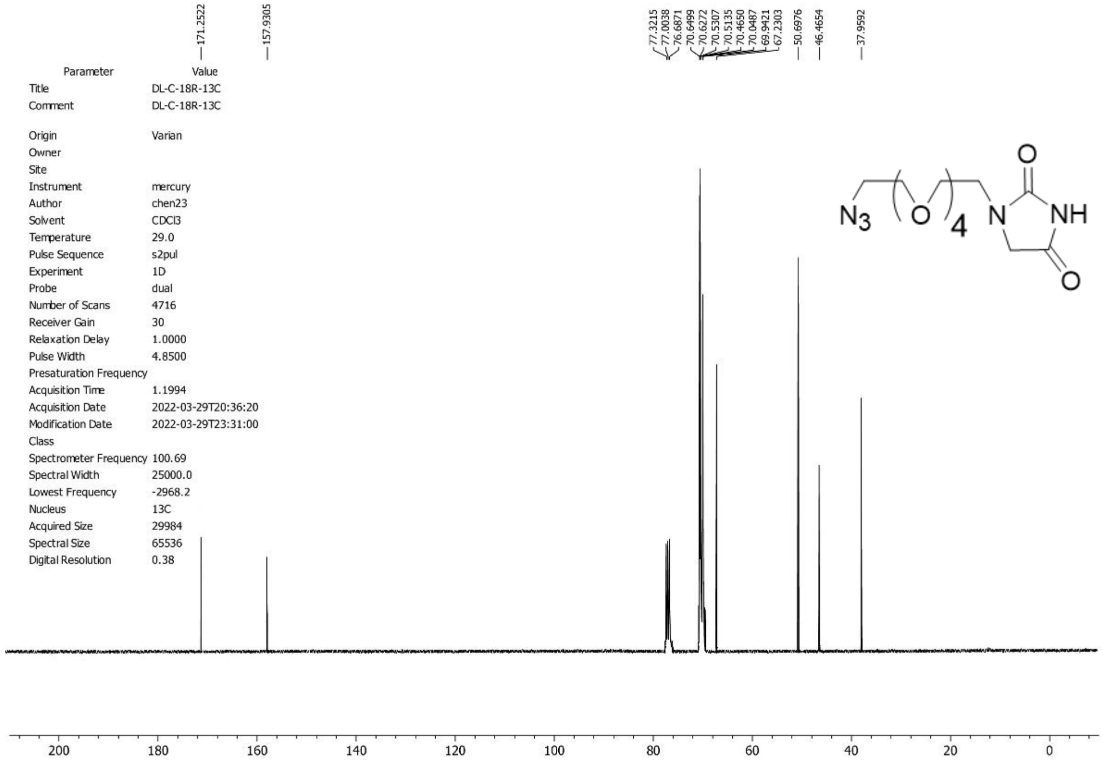

$^{13}$C NMR spectrum of compound **8b**

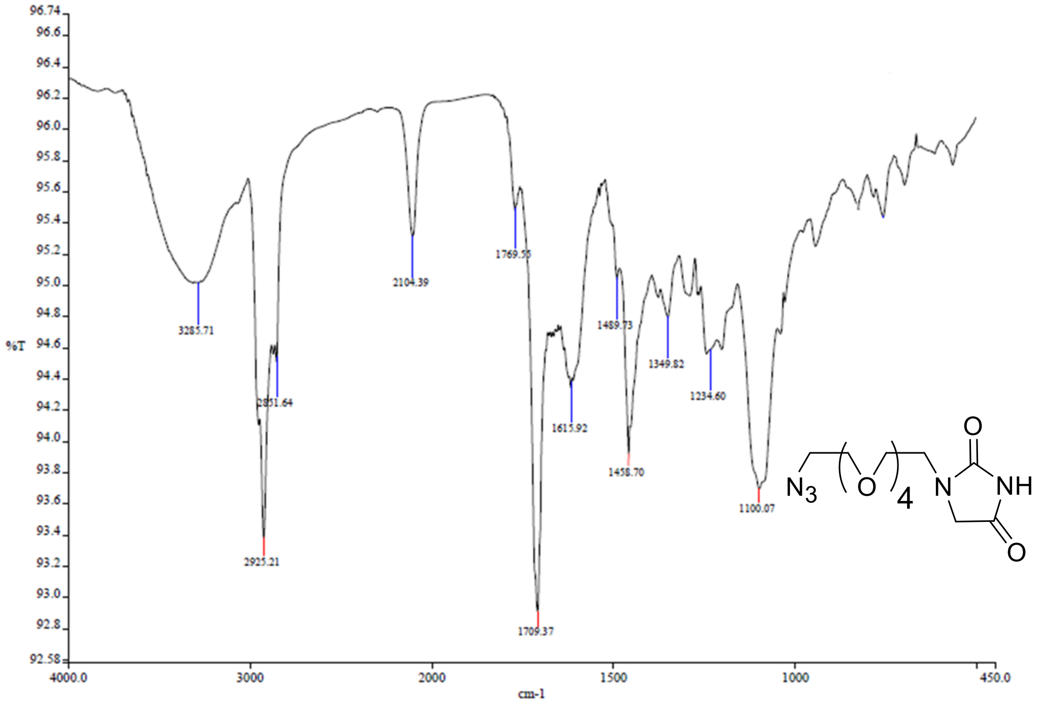


IR spectrum of compound **8b**

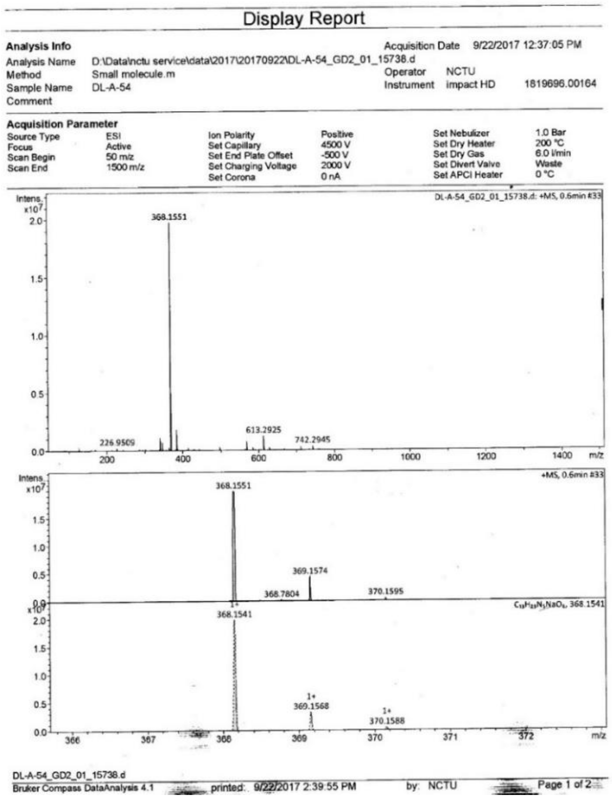


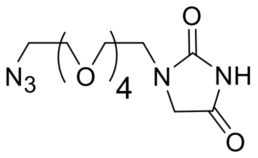


Mass spectrum of compound **8b**

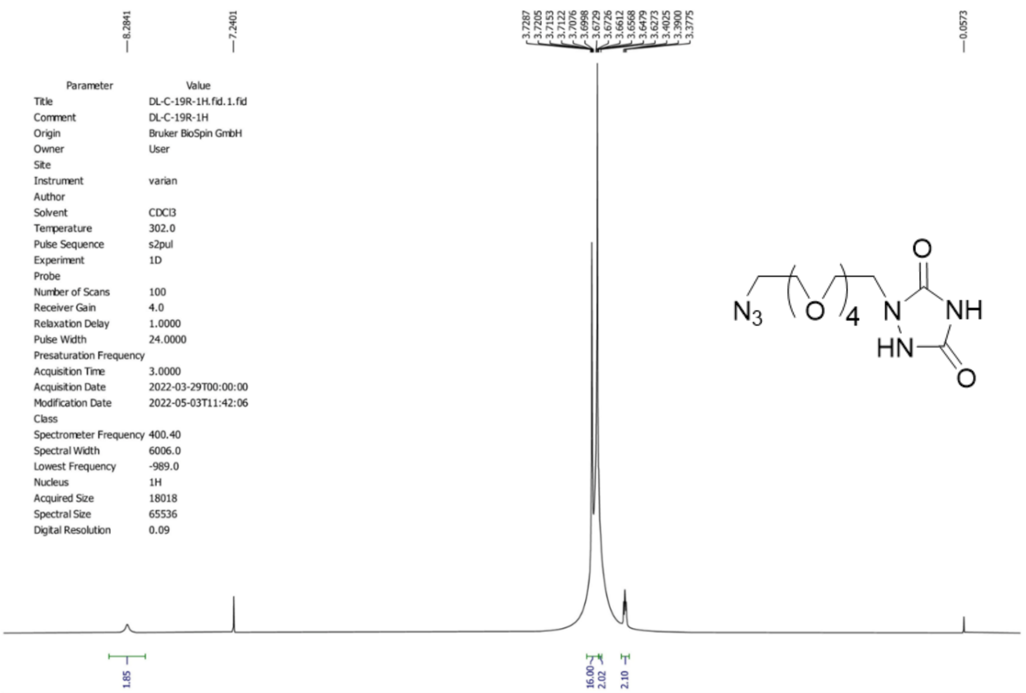

$^{1}$H NMR spectrum of compound **8c**

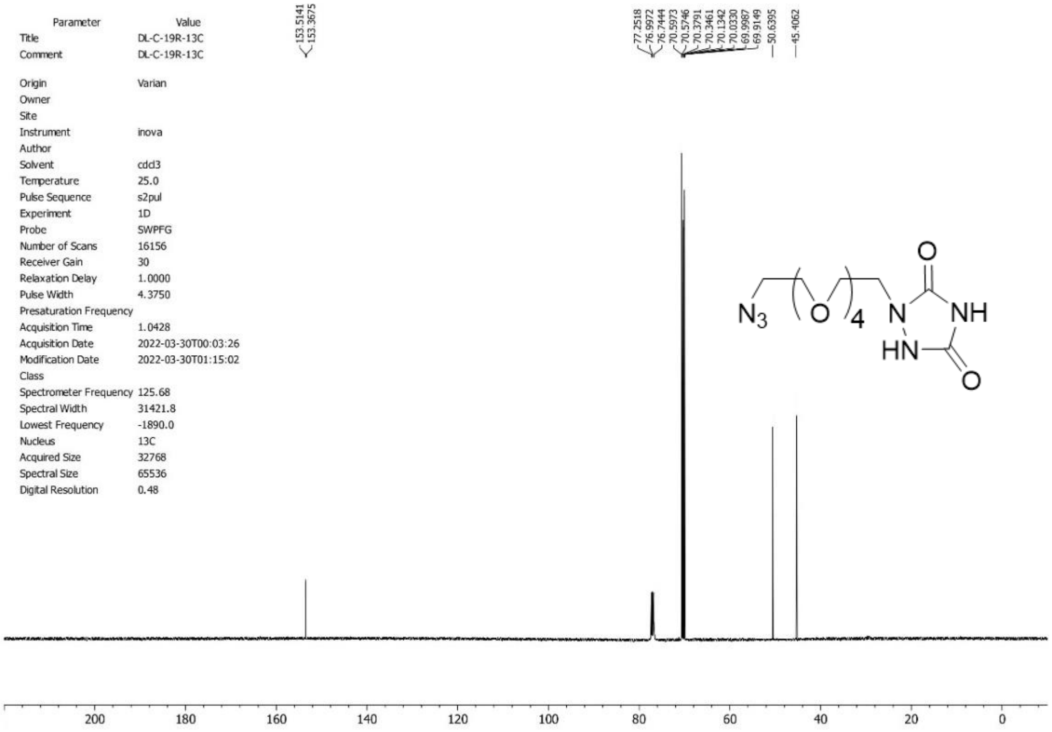

$^{13}$C NMR spectrum of compound **8c**

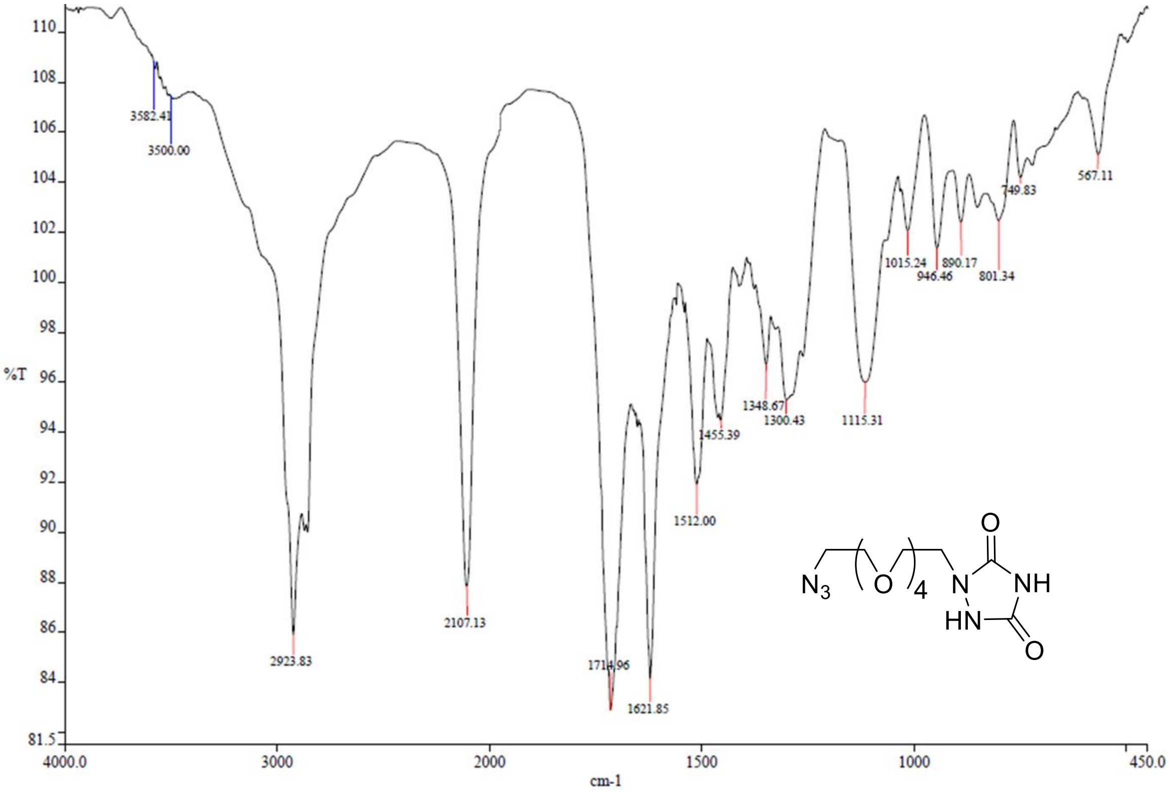


IR spectrum of compound **8c**

Data:DL-A-231-1
Comment:
Description:
Ionization Mode:ESI+
History:Average(MS[1] 0.53..0.58)

Acquired:8/9/2018 12:00:25 PM
Operator:AccuTOF
m/z Calibration File:20180525TFANa_...
Created:8/9/2018 2:23:06 PM
Created by:

Charge number:1
Tolerance:20.00[ppm], 10.00 .. 15.00[...
Unsaturation Number:-1.5 .. 20.0 (Frac...
Element:$^{12}$C:12 .. 12, $^{1}$H:0 .. 23, $^{79}$Br:0 .. 0, $^{35}$Cl:0 .. 0, $^{19}$F:0 .. 0, $^{14}$N:6 .. 6, $^{16}$O:6 .. 6, $^{32}$S:0 .. 0

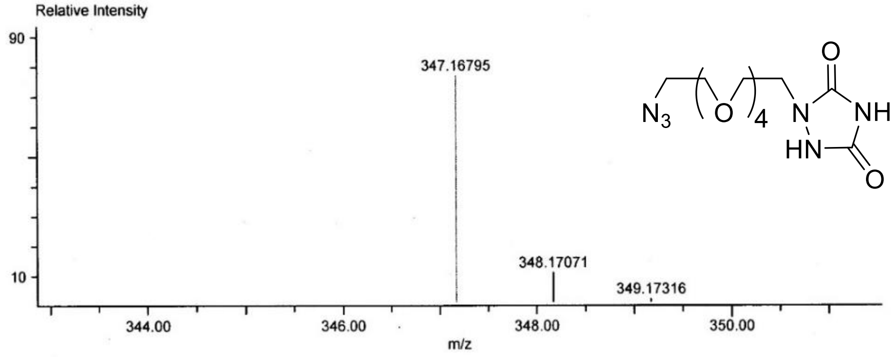


| Mass | Intensity | Calc. Mass | Mass Difference [mDa] | Mass Difference [ppm] | Possible Formula |
|---|---|---|---|---|---|
| 347.16795 | 105208.49 | 347.16791 | 0.04 | 0.11 | $^{12}C_{12}{}^{1}H_{23}{}^{14}N_6{}^{16}O_6$ |

Mass spectrum of compound **8c**

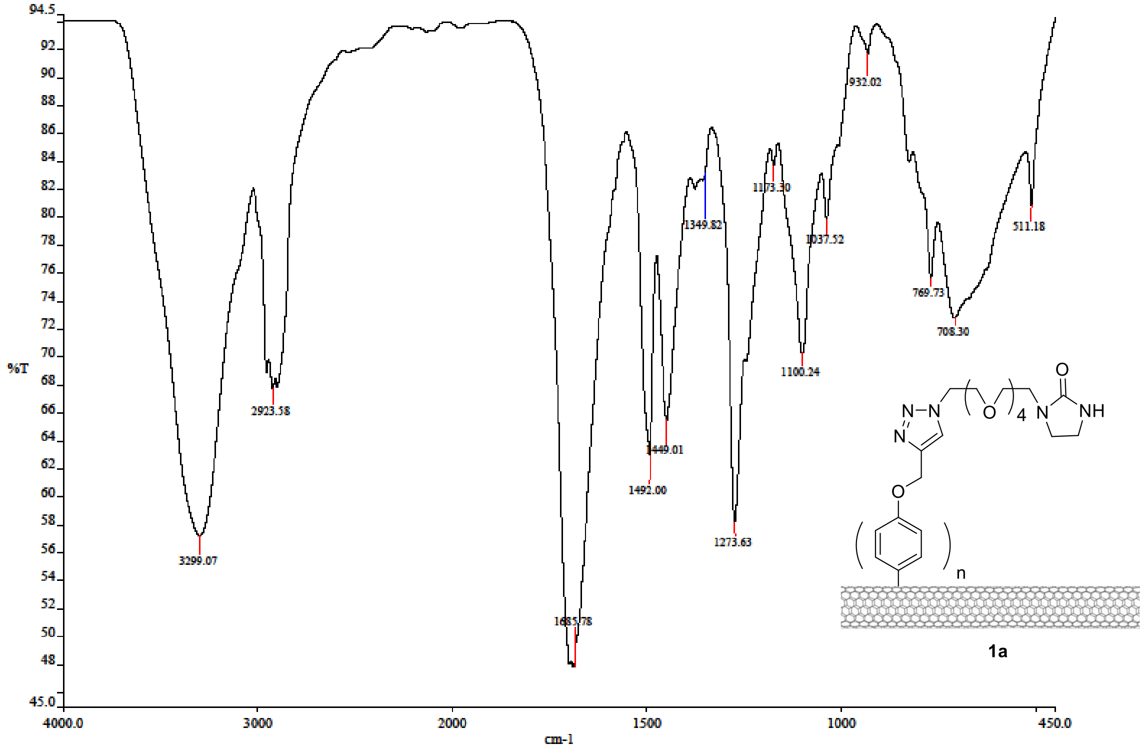


IR spectrum of compound **1a**

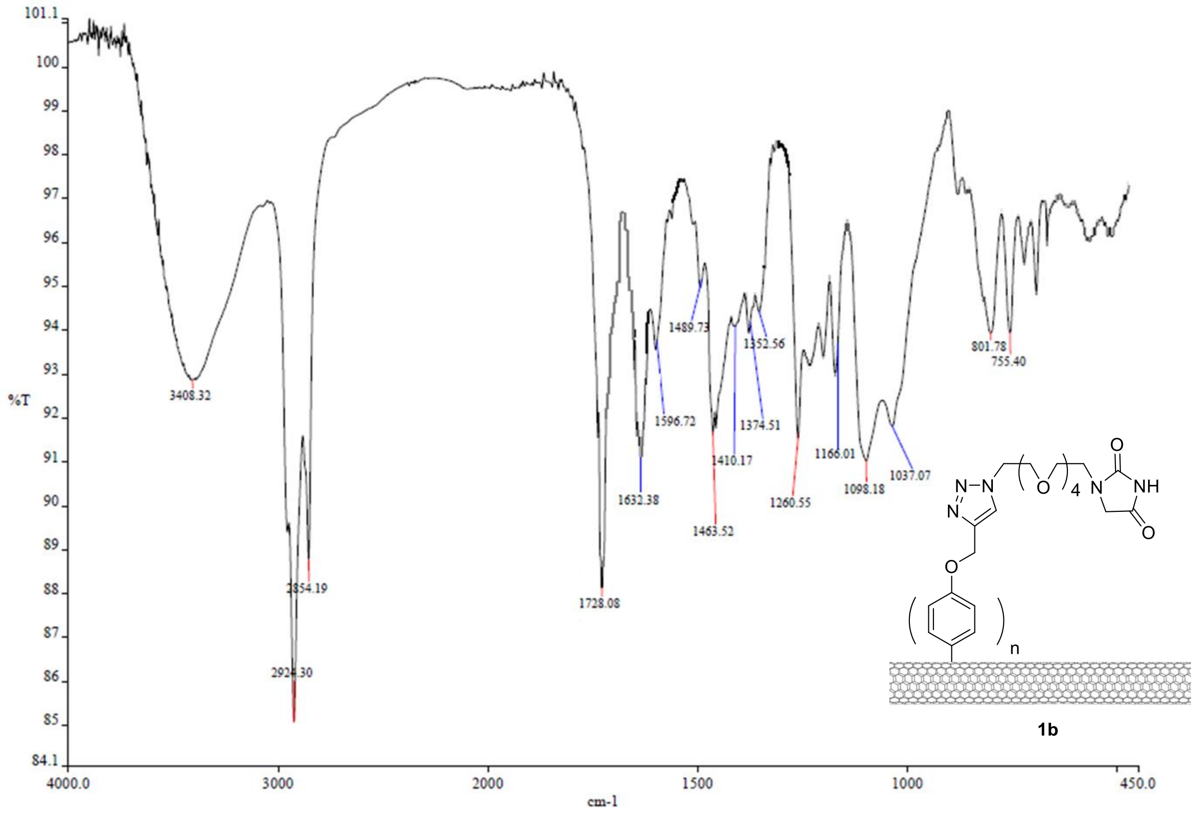


IR spectrum of compound **1b**

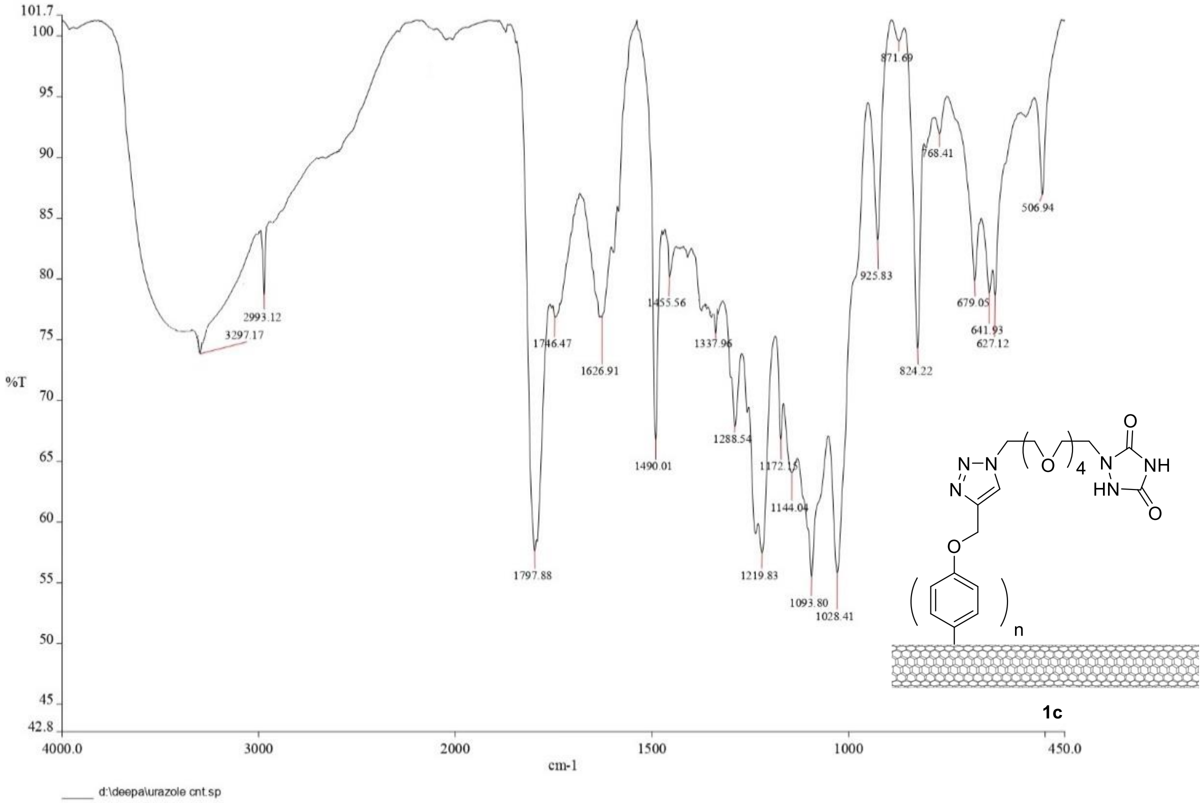


IR spectrum of compound **1c**
